# Objective identification of residue ranges for the superposition of protein structures

**DOI:** 10.1186/1471-2105-12-170

**Published:** 2011-05-18

**Authors:** Donata K Kirchner, Peter Güntert

**Affiliations:** 1 Institute of Biophysical Chemistry, Center for Biomolecular Magnetic Resonance, and Frankfurt Institute for Advanced Studies, Goethe University Frankfurt am Main, Max-von-Laue-Str. 9, 60438 Frankfurt am Main, Germany; 2 Frankfurt Institute for Advanced Studies, Goethe University Frankfurt am Main, Ruth-Moufang-Str. 1, 60438 Frankfurt am Main, Germany; 3 Graduate School of Science, Tokyo Metropolitan University, Hachioji, Tokyo, Japan

## Abstract

**Background:**

The automation of objectively selecting amino acid residue ranges for structure superpositions is important for meaningful and consistent protein structure analyses. So far there is no widely-used standard for choosing these residue ranges for experimentally determined protein structures, where the manual selection of residue ranges or the use of suboptimal criteria remain commonplace.

**Results:**

We present an automated and objective method for finding amino acid residue ranges for the superposition and analysis of protein structures, in particular for structure bundles resulting from NMR structure calculations. The method is implemented in an algorithm, CYRANGE, that yields, without protein-specific parameter adjustment, appropriate residue ranges in most commonly occurring situations, including low-precision structure bundles, multi-domain proteins, symmetric multimers, and protein complexes. Residue ranges are chosen to comprise as many residues of a protein domain that increasing their number would lead to a steep rise in the RMSD value. Residue ranges are determined by first clustering residues into domains based on the distance variance matrix, and then refining for each domain the initial choice of residues by excluding residues one by one until the relative decrease of the RMSD value becomes insignificant. A penalty for the opening of gaps favours contiguous residue ranges in order to obtain a result that is as simple as possible, but not simpler. Results are given for a set of 37 proteins and compared with those of commonly used protein structure validation packages. We also provide residue ranges for 6351 NMR structures in the Protein Data Bank.

**Conclusions:**

The CYRANGE method is capable of automatically determining residue ranges for the superposition of protein structure bundles for a large variety of protein structures. The method correctly identifies ordered regions. Global structure superpositions based on the CYRANGE residue ranges allow a clear presentation of the structure, and unnecessary small gaps within the selected ranges are absent. In the majority of cases, the residue ranges from CYRANGE contain fewer gaps and cover considerably larger parts of the sequence than those from other methods without significantly increasing the RMSD values. CYRANGE thus provides an objective and automatic method for standardizing the choice of residue ranges for the superposition of protein structures.

## Background

Most proteins comprise structured and unstructured regions. It is important to identify these regions to meaningfully compare or analyze protein structures. The most commonly used similarity measure for three-dimensional structures are atomic root-mean-square deviation (RMSD) values, which are computed for all or a subset of atoms in two or more structures after their optimal superposition, which is the one that minimizes the RMSD value [1]. For instance, NMR protein structures are generally represented by a bundle of conformers that have been calculated starting from different randomized initial conformations using identical input data, and it has been proposed to represent also crystallographic structures by an ensemble of conformations [2]. The precision of an NMR protein structure is measured by the average RMSD value between the individual conformers and their average coordinates, computed after superimposing the conformers onto the first one. Both the superposition and the resulting RMSD values are strongly influenced by the choice of atoms that are included in the fit. Including unstructured parts of a structure yields large RMSD values that may obscure the presence of well-defined structured regions of the protein. It is therefore crucial to identify the structured regions. A (subjective) choice can be made by visually inspecting the structures, but for reasons of consistency, reproducibility and efficiency an automated method is highly desirable. In this paper we present a new method for this purpose that has several advantages over existing approaches.

Various methods have been proposed to identify well-defined regions of a protein on the basis of the atomic coordinates of an ensemble of structures, such as the bundle of conformers resulting from an NMR structure determination [3-10]. Ordered regions can be identified by analyzing the local structure, for example by applying a cutoff on angular order parameters for the backbone torsion angles ϕ and ψ [11]. Ordered regions found by these strictly local approaches cannot necessarily be superimposed simultaneously with a low RMSD value. Other methods aim at finding the part(s) of a protein structure that are sufficiently similar to each other within the ensemble. Some algorithms rely on the distance variance matrix with elements *D*_*ij *_= σ(*d*_*ij*_)^2^, where σ(*d*_*ij*_) is the standard deviation of the distance between the atoms *i *and *j*, computed over all structures in the comparison [3-6]. Small matrix elements indicate that the position of the corresponding groups of atoms is similar in all members of the structure bundle. Another approach, implemented in the molecular graphics program MOLMOL [12], superimposes the structures with the current set of atoms (starting with all atoms or a user-defined subset), and then discards in each step the atoms from the residue with the largest global displacement, until either the RMSD falls below a maximally acceptable value or a minimal number of residues is reached. In the field of protein structure prediction [13], two algorithms, LCS ("longest continuous segments") and GDT ("global distance test"), have been established for detecting regions of local and global structure similarities [14]. The LCS procedure finds the longest continuous segment of residues that can fit under a given RMSD cutoff. The GDT algorithm searches for the largest (not necessarily continuous) set of residues that deviate by no more than a specified distance cutoff [14]. Results are reported as the percentage of residues under a given distance cutoff. A popular measure is the "GDT total score", *GDT_TS *= (*P*_1 _+ *P*_2 _+ *P*_4 _+ *P*_8_)/4, where *P*_*d *_is the fraction of residues that can be superimposed under a distance cutoff of *d *Å, which reduces the dependence on the choice of the cutoff by averaging over four different distance cutoff values.

## Results and Discussion

### The CYRANGE algorithm

**Figure 1:**
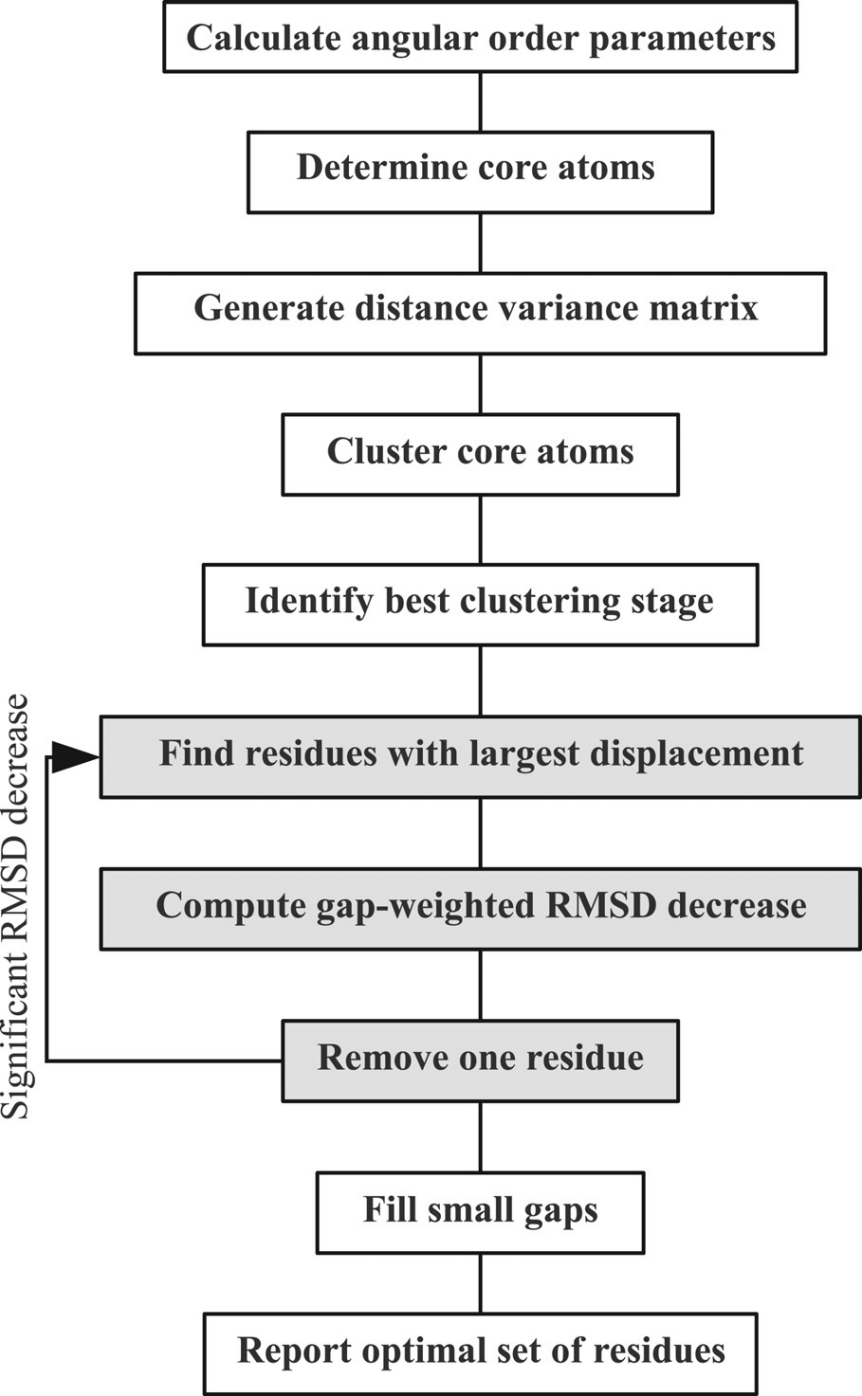
**Flowchart**. Flowchart of the CYRANGE algorithm for finding residue ranges for the global superposition of protein structures.

The CYRANGE algorithm (Figure 1) yields residue ranges for the superposition of protein structures with the same sequence. The algorithm has been designed (i) to find residue ranges that are suitable for the global superposition of protein domains (rather than detecting local order), (ii) to provide simple residue ranges with no or only a small number of gaps, (iii) to include as many residues as reasonably possible, (iv) to be applicable without change to structure bundles of high and low precision, (v) to be applicable to multi-domain proteins (without input specification of the domain boundaries), (vi) to handle symmetric multimers and protein complexes, and (vii) to work with a single set of parameters for all proteins. CYRANGE requires as input the Cartesian coordinates of two or more structures and consists of two main steps, domain identification and residue range determination for each domain.

#### Domain identification

Domain identification follows the approach used in the NMRCORE [6] and NMRCLUST [5] algorithms. Similar ideas have been used earlier [3,4,7,15]. Dihedral angle order parameter values of torsion angles from all conformers are computed. A cutoff value is calculated from the order parameter list and applied to select the torsion angles that will be used to identify the "core atoms". The core atoms are located in locally well-defined regions of the structure bundle, and are therefore potentially involved in domains that can be superimposed with low RMSD. The variances of the intra-conformer distances between all core atoms are used to cluster the core atoms, which eventually yields the single or multiple domains present in the structure bundle.

##### Angular order parameter calculation

Dihedral angle order parameters [11] are calculated from all rotatable dihedral angles (except the peptide bond dihedral angle ω). For a given torsion angle, the angular order parameter *S *with 0 ≤ *S *≤ 1 is given by

where the sum runs over the values θ_*k *_of a torsion angle in all *N *structures in the comparison. The higher the local order the higher the value of *S*.

If angular order parameters are computed from only the dihedral angles φ, ψ, and χ^1 ^the final results are largely identical to those obtained with the approach described above (see Results). Using all rotatable dihedral angles was given precedence as this approach yields a larger number of core atoms, and thus a larger base for clustering, while still excluding atoms from severely disordered parts of the protein.

##### Core atom determination

The set of core atoms consists of the C^α ^atoms of those residues that contain at least one well-ordered torsion angle with an angular order parameter *S *>*S*^cut^. We did not want to impose a fixed cutoff value *S*^cut ^because the degree of order within structure bundles is different in each case. Instead, the cutoff value *S*^cut ^is chosen as the angular order parameter value *S*_*i *_of the torsion angle *i *that maximizes the quantity

Here, *s *denotes the total number of torsion angles for which angular order parameters are calculated, *S*^max ^and *S*^min ^are the maximal and minimal angular order parameter values, and *r*_*i *_∈ {1,...,*s*} is the rank of the torsion angle *i *in an ordered list of the angular order parameter values (e.g. the torsion angle with the smallest *S*_*i *_has rank *r*_*i *_= 1, the torsion angle with the largest *S*_*i *_has rank *r*_*i *_= *s*). We denote the number of core atoms by *C*. Plots of *Q*_*i *_as a function of the order parameter rank *i *for various proteins (Additional File 1) show in all cases a clear maximum and the absence of distant, comparably high local maxima.

We found that using only C^α^s is sufficient for reliable domain identification. Additional core atoms merely slowed down the calculations. We also attempted to simply use all C^α ^atoms in the following clustering. This simple approach, however, was less reliable in domain identification than the present one based on angular order parameters.

##### Distance variance matrix

The variance *V*_*ij *_of the intra-conformer distance between any two core atoms *i *and *j *is calculated over all *N *members of the structure bundle,

where *d*_*ijk *_denotes the distance between atoms *i *and *j *in conformer *k*.

##### Core atom clustering

To determine the residues that belong to the same domain the core atoms are clustered using an agglomerative hierarchical clustering algorithm. At the outset (clustering stage 1) each core atom forms a cluster of its own. At each subsequent stage of clustering two clusters are merged, until at the end there remains a single cluster containing all core atoms. Hence there are as many clustering stages as there are core atoms. In each stage of clustering the two nearest neighbour clusters are identified and merged. All other clusters remain unchanged and are simply propagated to the next stage. The nearest neighbour clusters are defined by Ward's method as the two clusters that yield, after merging, the lowest intra-cluster *V*-value variance of all possible two-cluster combinations. The intra-cluster *V*-value variance is computed as the variance of the *V*_*ij *_values for all atoms *i *and *j *in the merged cluster, or, if the merged cluster contains only two atoms, by the corresponding single *V*_*ij *_value.

##### Identification of the best clustering stage

At each of the clustering stages *i *= 2, ..., *C *the average cluster spread

is calculated, where the sum runs over all clusters with more than one member, *c*_*i *_is the total number of core atoms from all clusters with more than one member, and RMSD_*j *_is the average over all structures of the RMSD to the mean coordinates for the backbone atoms N, C^α^, and C' of the residues given by the core atoms in cluster *j*. All RMSD values are calculated using singular value decomposition [16]. Low cluster spreads indicate high intra-cluster homogeneity, i.e. atom pairs with a similar degree of inter-atom distance variation are likely to belong to the same structural unit within the protein. A low number of clusters points to no artificial division of domains having occurred.

To determine the optimal clustering stage, the quantity

is calculated for the clustering stages *i *= 2, ..., *C*. *A*^min ^and *A*^max ^are the minimum and maximum average cluster spread values, respectively, and *n*_*i *_is the number of clusters at stage *i*, including single-element clusters.

The clustering stage with the lowest *P *value is chosen as the optimal clustering stage, i*, provided that the average number of core atoms in a cluster at stage i* exceeds one eighth of the total number of core atoms, rounded up to the nearest integer. In the calculation of this average cluster size only those clusters are considered which contain at least the minimal number of elements (*μ*; see below) required for a cluster to be considered as a domain. If the average cluster size is too small, a new minimum value of *P *is determined in the restricted range *i *= i* + 1, ..., *C*. The procedure is repeated until the average number of cluster elements at the clustering stage with minimum *P *exceeds one eighth of the total number of core atoms. The minimum of *P*_*i *_as a function of the clustering stage *i *is sharply defined, usually at or near the last clustering stage (Additional File 2).

Each cluster at the optimum stage of clustering is considered as a domain, provided that it contains at least *μ *elements. By default, *μ *= 8.

The residue ranges corresponding to the identified domains are passed to the residue range determination procedure. First, however, these ranges are extended at all boundaries by *m *residues, so as not to restrict the range determination procedure to perhaps too narrow a starting range. By default, *m *= 3.

#### Residue range determination

The determination of the residue range for each domain starts with the residues of a previously identified and subsequently extended domain. The algorithm proceeds by iteratively removing residues until the set of residues does not change in an iteration. The algorithm comprises the following 7 steps:

*(1) RMSD calculation: *Compute the RMSD value, *r*, for the backbone atoms N, C^α^, and C' of the current set of residues.

*(2) Removal of isolated residues: *If present, exclude from the set of residues those with two neighbours that do not belong to the current set of residues, and start a new iteration at step 1.

*(3) Find residues with largest displacements: *Among the selected residues find the one with the largest average displacement whose removal does not open a new gap in the selected residues. Similarly, find the residue with the largest average displacement whose removal opens a new gap in the selected residues. The average displacement corresponds to the distance between an atom in a given conformer and its mean position after optimal superposition of all conformers onto the first conformer in the RMSD calculation of step 1, averaged over all conformers and over the backbone atoms N, C^α^, and C' of a residue.

*(4) Compute gap-weighted RMSD decrease: *For the two residues found in step 3, compute the gap-weighted decrease of the RMSD value upon removing the residue from the set, Δ*r*^nogap ^= *r *- *r*^nogap ^and Δ*r*^gap ^= *γ *(*r *- *r*^gap^), where *r *is the RMSD value from step 1, *r*^nogap ^and *r*^gap ^are the RMSD of the selected residues after removing one of the residues from step 3, and *γ *is a dimensionless parameter that penalizes the opening of new gaps (if *γ *< 1).

*(5) Residue removal: *If the residue with the larger Δ*r *value fulfils the two conditions Δ*r *≥ δ ^abs ^*n*/*N *and Δ*r/r *≥ *δ *^rel ^*n*/*N*, remove it from the set of selected residues and start a new iteration at step 1. Here *n *and *N *denote the numbers of atoms included in the RMSD calculation of step 1 from the current residue and from all selected residues, respectively, and *δ *^abs ^and *δ *^rel ^are parameters for the minimally required absolute and relative RMSD decrease, respectively.

*(6) Retry residue removal: *If no residue was removed in step 5, find among *all *selected residues the residue whose removal yields the largest gap-weighted decrease of the RMSD value. If the conditions of step 5 are fulfilled for this residue, remove it from the set of selected residues and start a new iteration at step 1.

*(7) Fill small gaps: *If the set of selected residues contains small gaps of less than *g *(by default three) residues, "fill" these gaps by additionally selecting the residues in the gap.

Average displacements are calculated in step 3 to limit the number of RMSD calculations to two in step 4 and executing only rarely step 6 which requires an RMSD calculation for every selected residue.

In this paper, unless noted otherwise, we used *μ *= 8, *m *= 3, *γ *= 0.4, *δ *^abs ^= 1.6 Å, and *δ *^rel ^= *δ *+ 3.0/*M *with *δ *= 1.2, where *M *denotes the current number of selected residues, and *g *= 3. Smaller values of *γ *lead to fewer gaps. The choice of *δ *^rel ^was motivated by the observation of the relative RMSD decrease values for randomly disordered structures. The increase of *δ *^rel ^for small numbers of selected residues ensures the termination of the algorithm.

#### Output

The CYRANGE method yields for each of the domains it identified the residue range(s) for superposition, the number of residues therein, and the average RMSD value to the mean coordinates for the backbone atoms in the residue range(s) for superposition. If the input consists of only two structures, the RMSD to the mean coordinates is equal to half the RMSD between the two structures. The CYRANGE algorithm is freely available as a web service and a stand-alone program at http://www.bpc.uni-frankfurt.de/cyrange.html. It has also been implemented in the software package CYANA [17,18].

### Application of CYRANGE to 37 proteins

**Figure 2:**
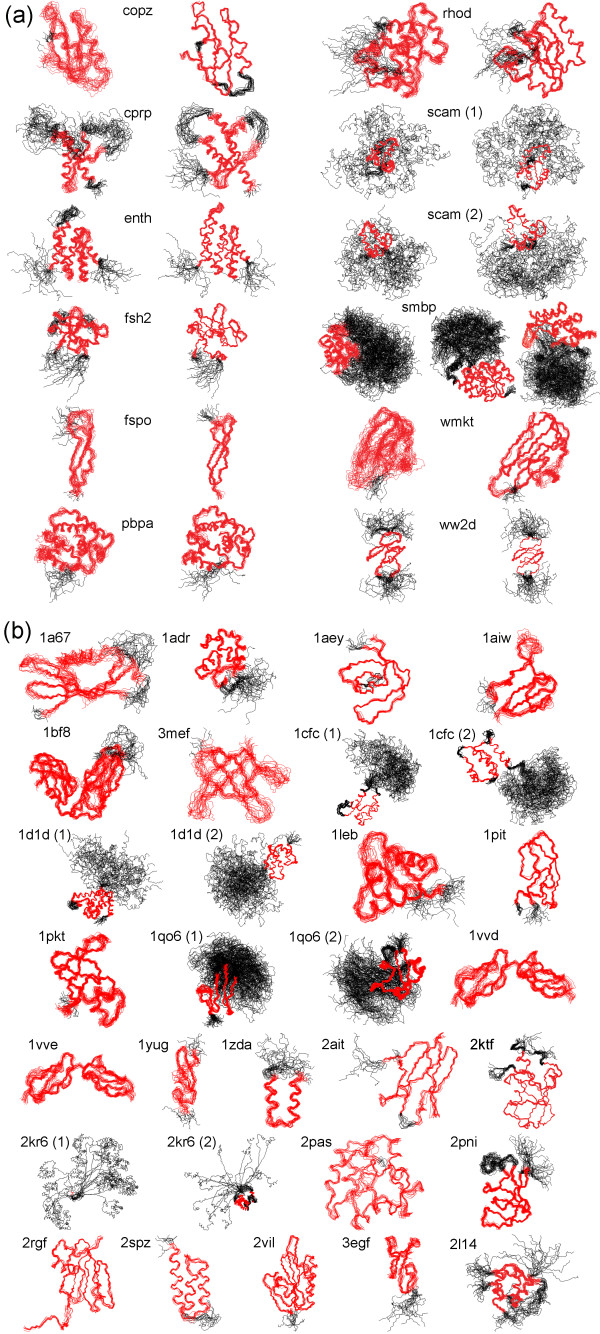
**Structure superpositions**. Structure bundles superimposed for minimal backbone RMSD of the CYRANGE-determined residue ranges, indicated in red. Other residues are in black. Separate superpositions are shown for each domain. (**a**) 11 proteins for which low-precision (left) and high-precision (right) NMR structures are available (see Methods). (**b**) 26 proteins, 23 of which had been used earlier for evaluating the FindCore algorithm.

**Figure 3:**
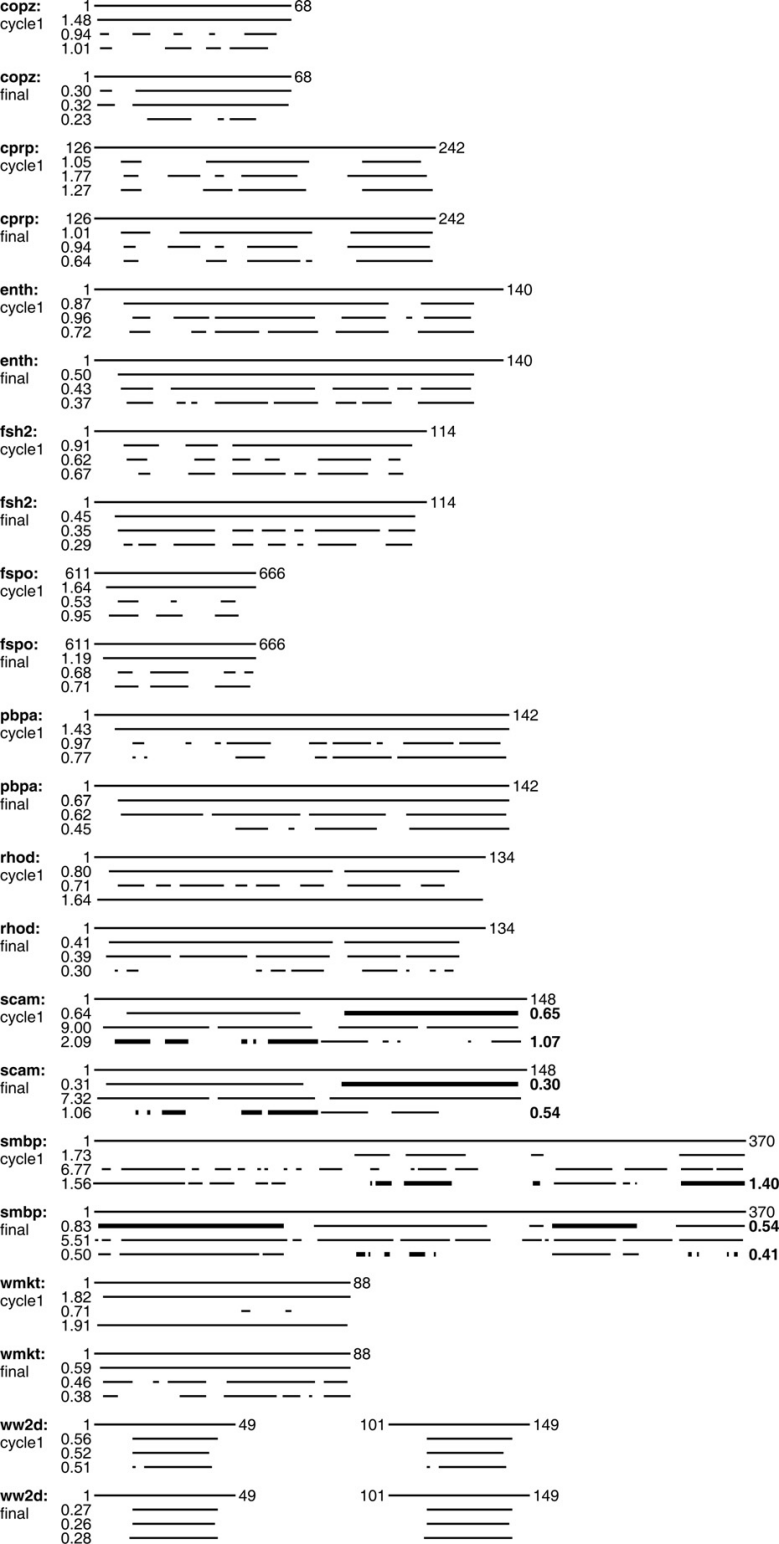
**Residue ranges for high- and low-precision NMR structures**. Comparison of residue ranges found by the CYRANGE, PSVS, and FindCore methods for 11 proteins for which low-precision (cycle1) and high-precision (final) NMR structures are available (see Methods). For each protein, the top line represents the complete amino acid sequence with the first and last residue labelled. Below are the residue ranges obtained from CYRANGE, PSVS, and FindCore. The corresponding RMSD values are indicated in Å. Multiple domains are distinguished by thin and thick lines.

**Figure 4:**
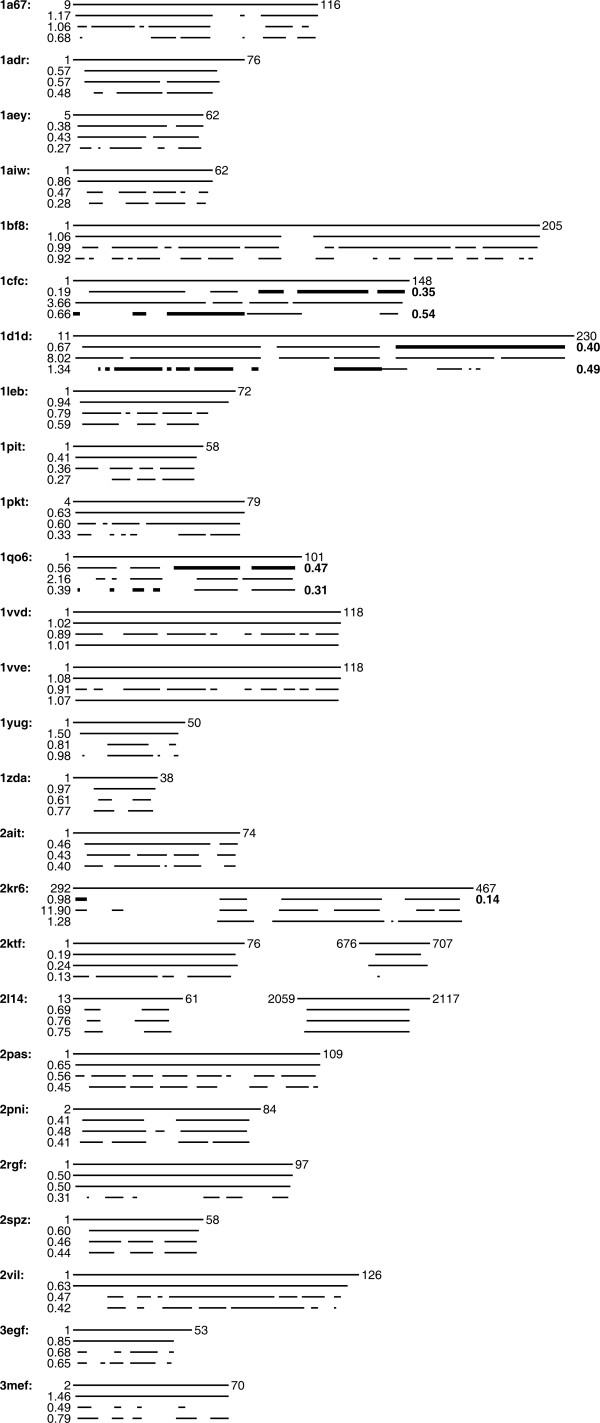
**Residue range comparison**. Comparison of residue ranges found by the CYRANGE, PSVS, and FindCore methods for 26 proteins. See Figures 2b and 3 for details.

The residue ranges determined by CYRANGE for 37 proteins are visualized in the 3D structure bundles of Figure 2 and summarized in Figures 3 and 4. Details regarding the proteins used are given in the Methods section. The method provided results that make sense by visual inspection in that ordered regions were correctly identified, the global structure superpositions based on the CYRANGE residue ranges allow a clear presentation of the structure, and unnecessary small gaps within the selected ranges are absent. CYRANGE yielded similar results for the corresponding low- and high-precision structure bundles of the 11 proteins of Figures 2a and 3, except that in enth and fsh2 additional loops, which are disordered only in the low-precision structure, were excluded, and that in the low-precision structure of smbp the second domain was not identified. For copz, a loop was excluded from the residue range for the high-precision structure that had been included for the low-precision structure because the residues are more uniformly disordered in the low-precision structure than in the high-precision one. The latter exhibited a higher standard deviation of the RMSD per-residue than the low-precision structure. This shows that the CYRANGE method gives meaningful results also in the challenging case of low-quality structures where the distinction between well-defined and ill-defined regions becomes blurred. For the protein 2kr6, the CYRANGE method correctly identified the two structural domains despite the small size of the isolated helix (Figures 2b and 4). The two protein-protein complexes, 2ktf and 2l14, did not pose any particular problems.

The CYRANGE results shown in Figures 2, 3, 4 have been obtained using the same parameter set for all proteins. We analyzed the influence of different values of the parameters on the resulting sequence coverage and RMSD for nine different protein structure bundles (Additional Files 3, 4, 5, 6, 7, 8). Each of the parameters *μ, m*, *δ*, *δ *^abs^, *γ*, and *g *was varied while keeping the other parameters at their default values. Varying the minimal cluster size *μ *in the range of 6-10 residues (Additional File 3), the domain boundary extension *m *in the range of 1-5 residues (Additional File 4), or the minimal gap width *g *in the in the range of 1-5 residues (Additional File 5) did not change the CYRANGE residue ranges for these 9 proteins, apart from the cases of 1zda and the low-precision structure of copz, where raising *m *led to minute changes in sequence coverage. Variation of the parameter *δ *for the relative RMSD decrease in step 5 of the residue range determination in the range 0.5-4 led to changes only for the already mentioned low-precision smbp structure (Additional File 6). Higher values of the corresponding absolute RMSD decrease parameter *δ *^abs ^led to slight increases of the sequence coverage and the RMSD for some of the proteins (Additional File 7). Similarly, a decrease of the sequence coverage and the RMSD were observed when increasing the gap penalty parameter *γ *from 0 (no gaps allowed) to 4 (gaps favoured) (Additional File 8). Thus the results from CYRANGE do not critically depend on the choice of these parameters but can, if desired, be guided towards a smaller or larger number of gaps or selected residues. The default values of the parameters appear to be appropriate for almost all proteins. Only the gap penalty parameter *γ *may occasionally be adapted according to the emphasis put on simple residue ranges with no or few gaps. Meaningful values of *γ *lie between 0, which yields a residue range without gaps, and 1, which selects residues without concern for the number of gaps.

We also compared the residue ranges obtained by computing the angular order parameters at the outset of the algorithm for all rotatable dihedral angles or only for φ, ψ, and χ^1^, using default parameter settings. For all but 4 out of the 37 proteins in the test set the results were identical. In the 4 other cases the sequence coverage, and usually the RMSD value, were higher when order parameters were calculated for all dihedral angles. The difference in coverage ranged from about 1 to 27%, and the difference in RMSD amounted to between 0 and about 0.7 Å. The 4 structure bundles for which differences became apparent were 2kr6, 2spz, the low-precision structure of fspo, and the high-precision structure of smbp. In the case of 2kr6 the small second domain was not identified when only φ, ψ, and χ^1 ^order parameters were used. For smbp the differences amounted to only 1 or 2 residues per domain. For 2spz the CYRANGE version using only φ, ψ, and χ^1 ^order parameters unnecessarily identified 3 small domains instead of the single one reported by standard CYRANGE. For fspo a disordered loop was excluded if only φ, ψ, and χ^1 ^order parameters were employed. Exclusion of this loop does seem sensible; raising the value of *γ *in the standard CYRANGE will also bring about this result. Overall, both choices of dihedral angle order parameters yielded similar results. Where differences occurred, the results obtained with order parameters for all dihedral angles mostly corresponded better to the conclusions from visual inspection of the structure bundles.

The computation time requirements of the CYRANGE algorithm are insignificant. It took CYRANGE 0.9 s to calculate the residue ranges for pbpa (142 residues, one domain), 1.0 s for scam (148 residues, two domains), and 5.8 s for smbp (370 residues, two domains) on a 2.66 GHz Intel Core 2 processor.

### Comparison with PSVS and FindCore

We compared the residue ranges from CYRANGE with those determined by the default algorithm of the Protein Structure Validation Suite PSVS, and by the FindCore algorithm. The algorithm in PSVS [19] is widely used for NMR protein structure validation, and was chosen here as a representative of the straightforward determination of (locally) ordered residues. PSVS does therefore not attempt to identify structural domains. FindCore [4], determines residue ranges for global superposition and is able to identify multiple domains.

**Figure 5:**
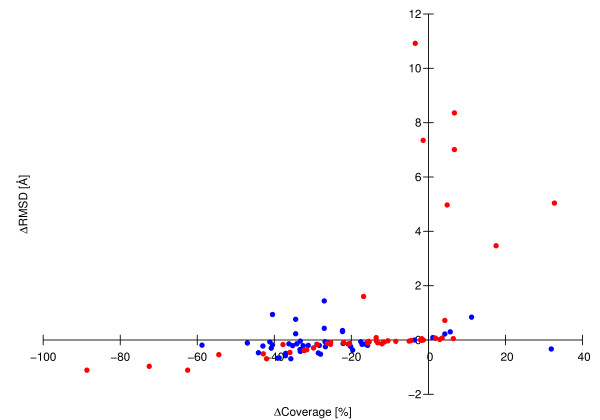
**RMSD and sequence coverage comparison**. RMSD and sequence coverage differences between the residue ranges found by PSVS (red) or FindCore (blue) and CYRANGE for the proteins shown in Figures 3 and 4. The coverage is the percentage of amino acid residues included in the residue ranges found by the different methods.

The residue ranges obtained with the three algorithms for 37 different proteins are shown in Figures 3 and 4, and the differences of the RMSD and sequence coverage relative to the CYRANGE results are shown in Figure 5. In the majority of cases, the residue ranges from CYRANGE contain fewer gaps and cover significantly larger parts of the sequence than those from PSVS and FindCore. Consequently, the RMSD values for the residue ranges identified by CYRANGE are often slightly higher than those from PSVS and FindCore. This, however, does not constitute a general rule. For instance, for the two-domain proteins 1cfc and 1d1d all CYRANGE domains simultaneously comprised more residues and showed lower RMSD values than those obtained from the other two algorithms (Figure 4). On average, the residue ranges reported by CYRANGE covered 85% of the sequences of these proteins and led to a backbone RMSD value of 0.77 Å, as compared to 67% coverage and 1.72 Å RMSD with PSVS, and 58% coverage and 0.73 Å RMSD with FindCore.

In the proteins with multiple domains both CYRANGE and FindCore found all domains except one. CYRANGE missed a domain in the low-precision smbp structure, FindCore in 2kr6. Figure 5 compares the RMSD and sequence coverage of the PSVS and FindCore results with those obtained by CYRANGE. Each data point represents one protein domain. Data points above/below the horizontal axis indicate cases where PSVS or FindCore yielded larger/smaller RMSDs than CYRANGE. Data points to the right/left of the vertical axis indicate cases where PSVS or FindCore yielded larger/smaller sequence coverage than CYRANGE. Most data points are found near the horizontal axis in the lower left quadrant. For these proteins CYRANGE covered typically between 10 and 50% more of the sequence with a small concomitant increase of the RMSD. Only a single data point, corresponding to the low-precision smbp structure, is located in the lower right quadrant, indicating a significantly smaller sequence coverage and higher RMSD by CYRANGE (because the algorithm failed to identify the separate domains of this two-domain protein). In all other cases of higher sequence coverage by PSVS or FindCore the greater number of selected residues resulted in larger, often much larger RMSDs. There are also some cases in which, especially with FindCore, simultaneously a smaller sequence coverage and a larger RMSD were found than with CYRANGE.

We have also correlated the sequence coverage and the RMSD values obtained by the three methods with the GDT total score, *GDT_TS*, that reports the average percentage of residues that can be superimposed under distance cutoffs of 1, 2, 4, and 8 Å [14]. Whereas there is a correlation between the sequence coverage by CYRANGE and the *GDT_TS *value, no such correlation is apparent for FindCore or PSVS (Additional File 9). The sequence coverage by FindCore is around 60% for all proteins except for four cases with nearly 100% sequence coverage, and independent from the *GDT_TS *value. The RMSD values do not correlate strongly with the *GDT_TS *values for any of the three methods (Additional File 10). This is not surprising because the *GDT_TS *measures the fraction of residues that can be superimposed reasonably well, whereas the RMSD reports how well a given subset of residues, which may comprise a smaller or larger part of the entire protein sequence, can be superimposed.

More detailed investigations into the differences between FindCore, PSVS, and CYRANGE results were performed, as the results, especially the number of gaps reported by the programs, vary considerably in some cases. Two examples of CYRANGE reporting few or no gaps where FindCore and PSVS report a high number of gaps are the results obtained for the low-precision structure of pbpa, and for 1bf8. In the first case CYRANGE excluded one disordered chain terminus, but it did not exclude a loop which, by visual inspection, seems slightly less ordered than the rest of the domain. PSVS kept the helices, yet excluded from them small, not considerably disordered-looking segments. It also excluded the chain termini, one of which does not seem highly disordered. The FindCore result was similar. Here we additionally found that some very small portions of otherwise excluded stretches were reported to be part of the domain. From 1bf8 CYRANGE excluded one highly disordered loop. PSVS also excluded this loop, alongside parts which, by visual inspection, did not appear to be considerably disordered. Again, the FindCore result was similar to the one attained by PSVS, but the rather ordered-looking sections excluded by FindCore were often somewhat larger.

### Application to all NMR structures in the PDB

**Figure 6:**
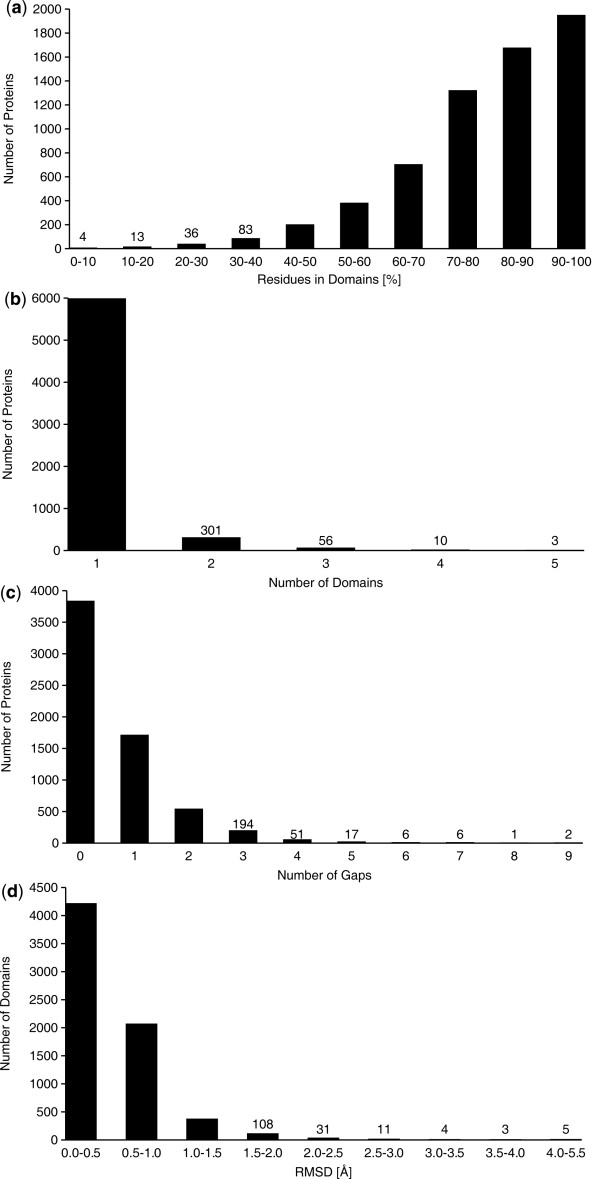
**Application to all NMR structures in the PDB**. Statistics of residue ranges determined with the CYRANGE algorithm for 6351 NMR protein structure bundles in the Protein Data Bank as of July 2010. (**a**) Percentage of residues in the residue range(s). (**b**) Number of domains. (**c**) Number of intra-domain gaps between the residue ranges. (**d**) RMSD values for the residue range.

On July 30, 2010 the PDB contained 6373 entries with protein structures determined by NMR that comprised at least 15 residues and for which a bundle of at least 5 conformers was available. We applied CYRANGE to all of these structure bundles, and obtained results for all but 22 files. In 4 cases no core atoms could be identified because the PDB files contained C^α ^positions only, in 1 case the residue range determination failed, and in 17 cases no domains were found. 14 of those proteins comprised less than 20 residues and often consisted of a single helix with one or two disordered tails. Of the remaining three structures two were highly disordered, and one, made up of 25 residues, again contained a single helix with disordered tails only. The results for the remaining 6351 files are summarized in Figure 6. A complete list of the residue ranges and RMSD values is available at http://www.bpc.uni-frankfurt.de/cyrange_pdb.html. On average, the residue ranges covered 80% of the residues of a protein, and there were 1.07 domains and 0.59 intra-domain gaps per protein.

For 95% of the proteins the CYRANGE residue ranges comprised more than 50% of all residues (Figure 6a). In only four cases (PDB IDs 1r5s, 2fft, 2kes, 2v93) the residue ranges included less than 10% of all residues. Visual inspection of these structure bundles revealed that the low percentage of selected residues is correct for the two structures 1r5s and 2fft, which consist of a single helix with long, disordered tails, whereas a larger residue range should have been selected for 2kes, which consists of a single α helix. The PDB entry 2v93 could not be handled properly because it combines multiple conformations within the individual conformers.

Figure 6b shows that in 94% of structures CYRANGE identified a single domain. Two domains were found in 5% of the structures, and in 69 cases (1%) CYRANGE found three or more (maximally 5) domains. In two cases (1zll and 2hyn) the structure was a pentamer. In the third case (2k27) visual inspection showed that the protein consists of two globular domains, which were correctly identified by CYRANGE with RMSDs of about 1.1 Å, and extended stretches at the chain termini and in the connection between the two globular domains, where CYRANGE identified three small "domains" of 14-17 residues with RMSDs of 1.23-1.96 Å.

For 60% of the structures CYRANGE determined a residue range without intra-domain gaps (Figure 6c). Four or more intra-domain gaps were identified in only about 1% of the structures, i.e. the CYRANGE algorithm selected whenever possible simple residue ranges with no or only very few gaps.

The distribution of the backbone RMSD values for the domains identified by CYRANGE (Figure 6d) indicates that 62% of all domains have an RMSD value below 0.5 Å. Less than 1% exhibit RMSD values above 2 Å, and only five domains reported by CYRANGE are severely disordered with RMSD values in the range of 4.0-5.5 Å, which appear to be largely ill-defined also by visual inspection. This shows that in almost all cases CYRANGE determined residue ranges that can be superimposed well. Considering the large number of domains, it cannot be excluded that in some cases more appropriate residue ranges could be identified by visual inspection or other methods. Nevertheless, the facts that the algorithm failed to provide a result for only 0.34% of the PDB entries and that for more than 99% of the domains CYRANGE yielded residue ranges with RMSDs below 2 Å and covering a significant fraction of the sequence indicate the usefulness of CYRANGE as a general tool for objective residue range determination.

## Conclusions

We introduced the CYRANGE algorithm for the determination of residue ranges for the superposition of protein structures and showed that it provides meaningful results for a large number of NMR protein structure bundles. The algorithm needs no adaptations of parameters for individual protein structures. CYRANGE is a tool for protein structure analysis that is available for easy integration in validation packages [19-23]. Although algorithms for the same purpose have been developed earlier [3-7,14], none of them has become a widely-used standard for experimentally determined protein structures, where, despite a clear need for standardization and automation, the manual selection of residue ranges or the use of suboptimal criteria remain commonplace. We hope CYRANGE to fill this gap.

## Methods

### Use of FindCore and PSVS

For comparison, also the Protein Structure Validation Software Suite (PSVS) [19] and the program FindCore [4] were employed to identify residue ranges and domains in our protein test set. The programs were used through the web portals http://psvs-1_4-dev.nesg.org (PSVS) and http://fps.nesg.org (FindCore).

With PSVS the default option 'ordered residues' was selected for the residue selection for analysis. The residues reported as "ordered" were taken as the residue ranges identified by PSVS. Note that with these options PSVS does not identify multiple domains.

With FindCore, the 'average structure' was selected as the reference structure, the analysis was based on 'standard amino acids', and only backbone atoms were used in domain identification. The calculations were also performed using all instead of only backbone atoms, with largely equivalent results (data not shown). FindCore reports the number of domains it identified, yet it does not unambiguously state the boundaries of the domains. Instead, the program provides a list of 'core residues'. When the program reported more than one domain, we manually attributed the core residues to the individual domains.

### Analyses of the results

PSVS and FindCore results were downloaded from the internet, and the output residue ranges were extracted from the source code of the downloaded web pages in an automated fashion. All reported RMSD values were calculated with CYANA for the backbone atoms N, C^α^, and C' in the reported residue ranges, and with respect to the mean coordinates. With FindCore the RMSD value of each identified domain was calculated separately; for PSVS all reported 'ordered residues' were used in the RMSD calculations, as no domain information is provided by PSVS. The program MOLMOL [12] was used to visualize structures.

### Determination of the average *GDT_TS *value of each structure bundle

The web server on http://proteinmodel.org/AS2TS/LGA/lga.html (parameter set -3 -o0 -d:4.0) was used for obtaining the *GDT_TS *values whose averages are shown in the Additional Files 9 and 10. From each structure bundle all possible conformer pairs consisting of the first conformer and each subsequent conformer were subjected to the calculation. The results were extracted from the web site in an automated fashion, and for each structure bundle the average of the individual *GDT_TS *values was computed.

### NMR protein structures

The performance of CYRANGE was assessed on the basis of the NMR structure bundles of the 11 proteins, for which the NMR solution structure had been determined earlier. We refer to these proteins by four-letter codes: copz [24], PDB 1CPZ; cprp [25], PDB 1U3M, enth [26,27], PDB 1VDY; fsh2 [28,29], PDB 1WQU; fspo [30], PDB 1VEX; pbpa [31], PDB 1GM0; rhod [32,33], PDB 1VEE; scam [34], PDB 1X02; smbp [34], PDB 2D21; wmkt [35], PDB 1WKT; ww2d [36], PDB 2DWV.

The proteins copz, cprp, enth, fsh2, pbpa, rhod and wmkt are proteins with a well-defined single-domain structure. The protein fspo has an unusual, less well-defined fold without regular secondary structure. The proteins scam and smbp are proteins with two domains connected by a flexible linker. The protein ww2d forms a symmetric dimer.

Two structure bundles were considered for each of these proteins: the final structure bundle, and the structure bundle obtained in the initial cycle 1 of automated NOE assignment and structure calculation [37] with CYANA [17], i.e. all structures were recalculated using the experimental chemical shift lists, NOESY peak lists, and possible additional torsion angle or hydrogen bond restraints. This enabled comparisons of the CYRANGE ranges for two structure bundles of different precision and quality for each of the proteins.

CYRANGE was also applied to a set of 26 NMR protein structures, 23 of which had been used earlier for evaluating the FindCore algorithm [4]. In addition, the protein 2kr6 was included as an example of a protein with a large domain and a flexibly connected small helix that constitutes a separate domain [38] and 2ktf and 2l14 were included as examples of protein-protein complexes. We refer to these 26 proteins by their PDB codes.

### Application to all NMR structures in the PDB

The entire set of NMR structures from the PDB [39] as available on July 30, 2010 was subjected to domain and residue range determination with the CYRANGE method, provided that the files contained at least five conformers and 15 amino acid residues.

## Authors' contributions

DK applied the method to protein structures and performed the statistical analysis. PG conceived of the study. Both authors designed the research, implemented computer algorithms, and wrote and approved the final manuscript.

## Supplementary Material

Additional file 1 **Dependence of *Q *on the order parameter rank**. The quantity *Q*_*i *_is plotted against the order parameter rank *i *for 9 different protein structure bundles. Click here for file

Additional file 2 **Dependence of *P *on the clustering stage**. The quantity *P*_*i *_is plotted against the clustering stage *i *for 9 different protein structure bundles. Click here for file

Additional file 3 **Dependence of CYRANGE results on the minimal cluster size parameter *μ***. The sequence coverage (red) and RMSD (blue) of the residue ranges determined by CYRANGE were plotted as a function of *μ *for 9 different protein structure bundles. The dotted vertical line indicates the default value, *μ *= 8. Where CYRANGE found two domains, the RMSD values of the individual domains are shown in light and dark blue. Click here for file

Additional file 4 **Dependence of CYRANGE results on the domain boundary extension parameter *m***. See Additional File 3 for details. Click here for file

Additional file 5 **Dependence of CYRANGE results on the minimal gap width *g***. See Additional File 3 for details. Click here for file

Additional file 6 **Dependence of CYRANGE results on the relative RMSD decrease parameter *δ***. See Additional File 3 for details. Click here for file

Additional file 7 **Dependence of CYRANGE results on the absolute RMSD decrease parameter *δ ***^**abs**^. See Additional File 3 for details. Click here for file

Additional file 8 **Dependence of CYRANGE results on the gap penalty parameter *γ***. See Additional File 3 for details. Click here for file

Additional file 9 **Correlation between the sequence coverage from CYRANGE, FindCore and PSVS, and the GDT total score, *GDT_TS***. Each data point represents a protein shown in Figures 3 and 4. The coverage is the percentage of amino acid residues included in the residue ranges found by the different methods. The *GDT_TS *value is defined by *GDT_TS *= (*P*_1 _+ *P*_2 _+ *P*_4 _+ *P*_8_)/4, where *P*_*d *_is the fraction of residues that can be superimposed under a distance cutoff of *d *Å. Click here for file

Additional file 10 **Correlation between the RMSD value for the residue ranges from CYRANGE, FindCore and PSVS, and the GDT total score, *GDT_TS***. Each data point represents one protein domain. See Additional File 9 for details. Click here for file
