# Supplementary figures and images for: Objective identification of residue ranges for the superposition of protein structures
Source: BMC Bioinformatics. 2011 May 18;12:170. doi: 10.1186/1471-2105-12-170 (PMC3120703; doi:10.1186/1471-2105-12-170)

**1bf8**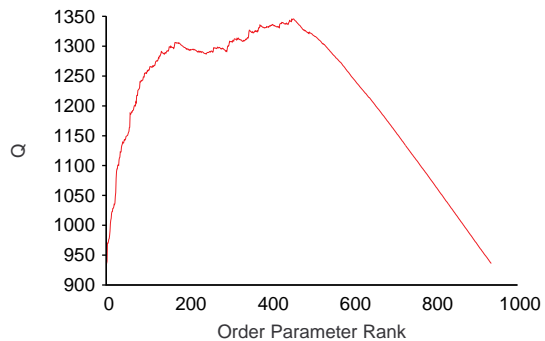**ww2d-cycle1**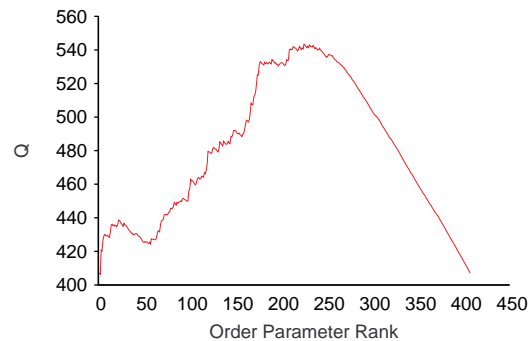**ww2d-final**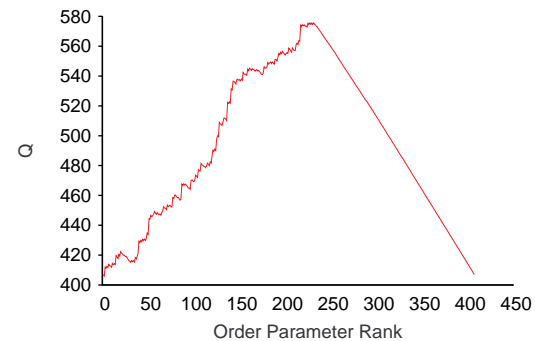**1cfc**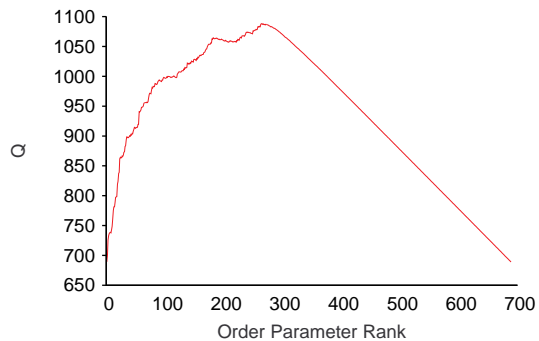**smbp-cycle1**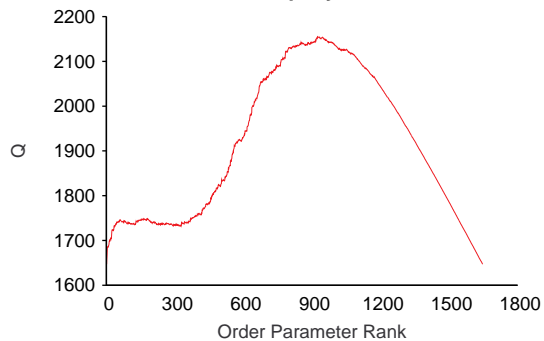**smbp-final**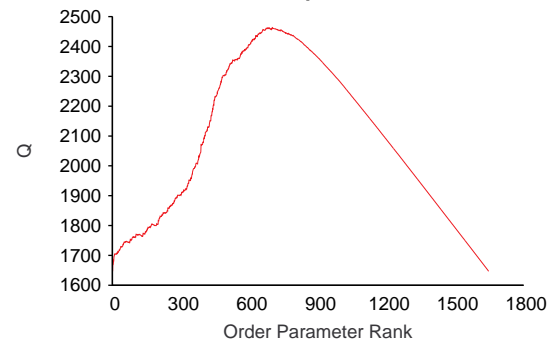**1zda**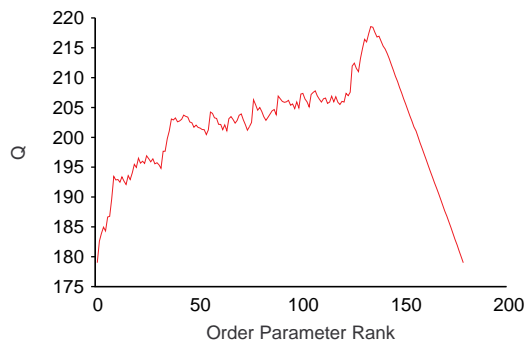**copz-cycle1**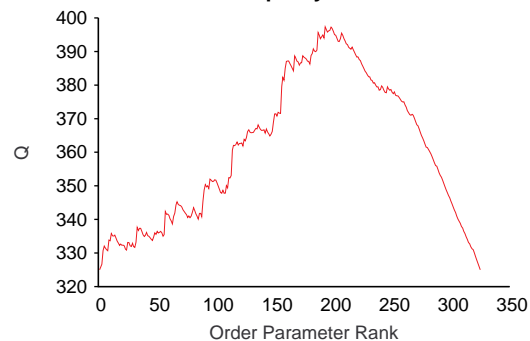**copz-final**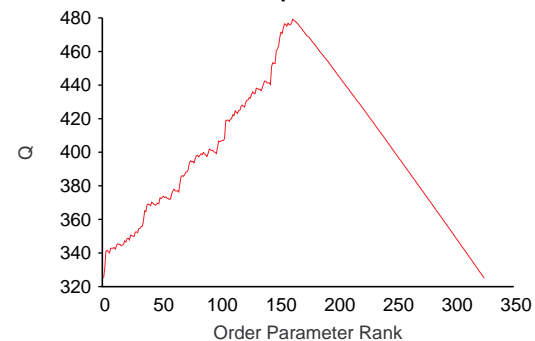

Supplement: Additional file 1 — Dependence of Q on the order parameter rank. The quantity Qi is plotted against the order parameter rank i for 9 different protein structure bundles. [file 1471-2105-12-170-S1.PDF]

**1bf8**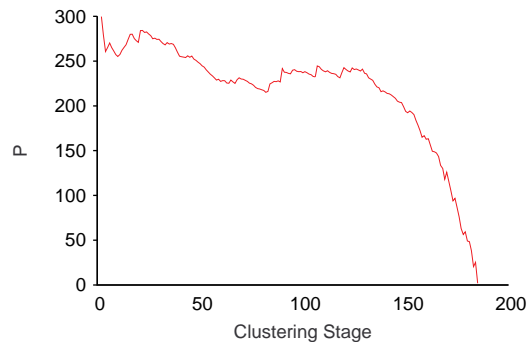**ww2d-cycle1**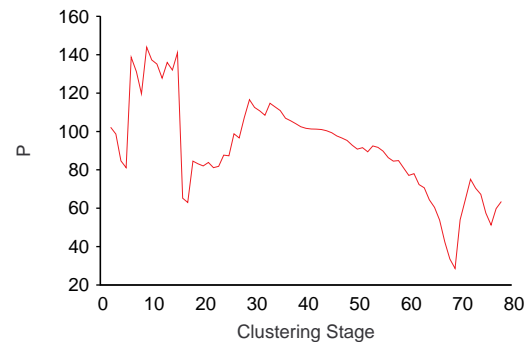**ww2d-final**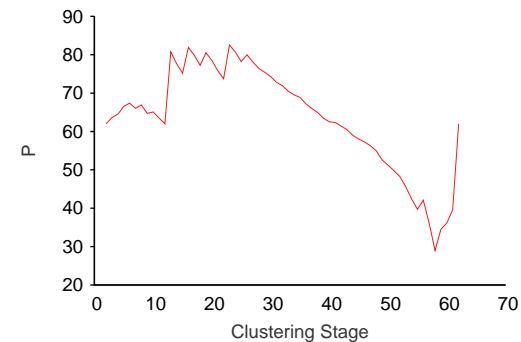**1cfc**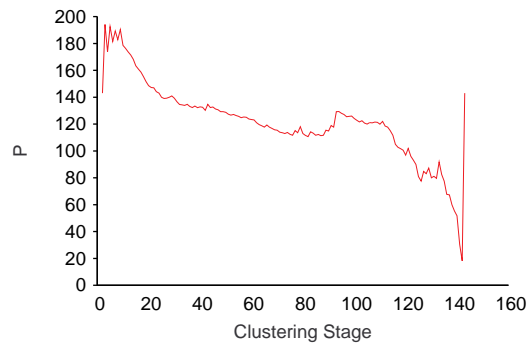**smbp-cycle1**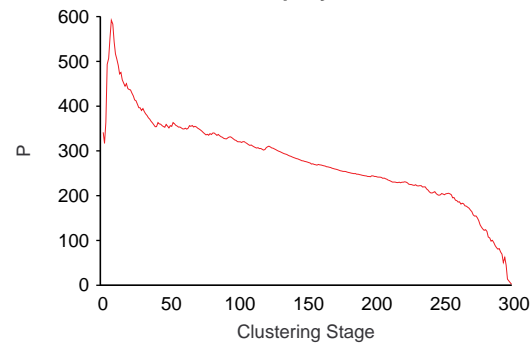**smbp-final**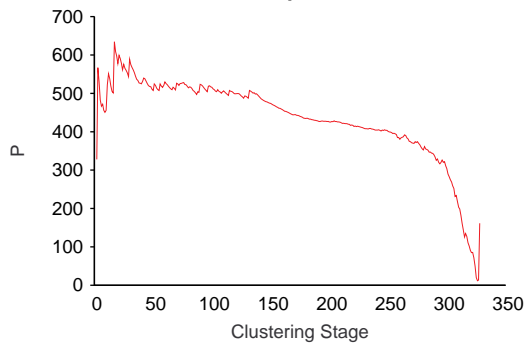**1zda**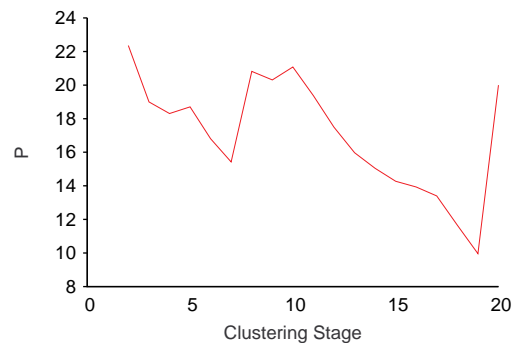**copz-cycle1**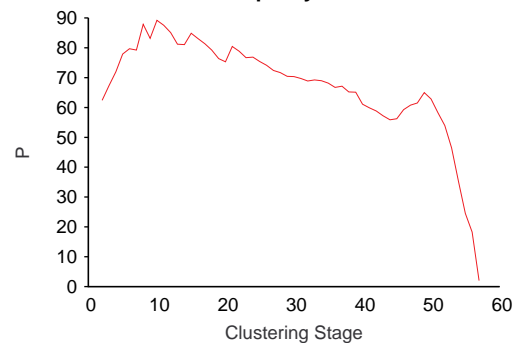**copz-final**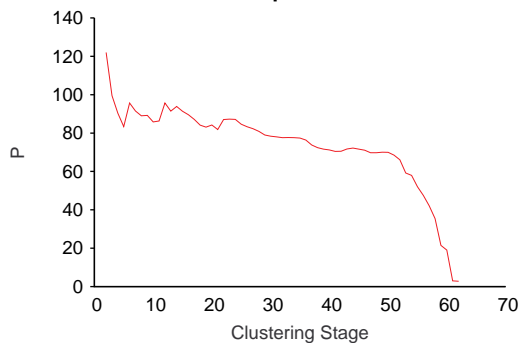

Supplement: Additional file 2 — Dependence of P on the clustering stage. The quantity Pi is plotted against the clustering stage i for 9 different protein structure bundles. [file 1471-2105-12-170-S2.PDF]

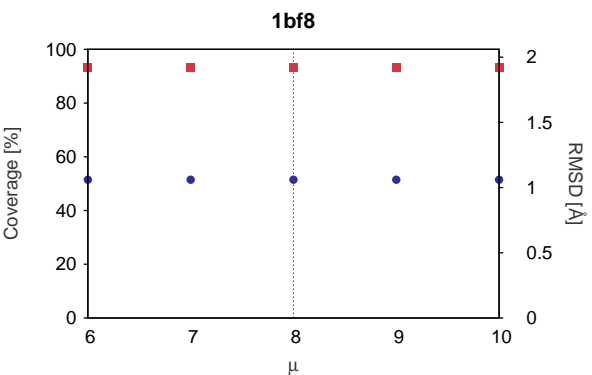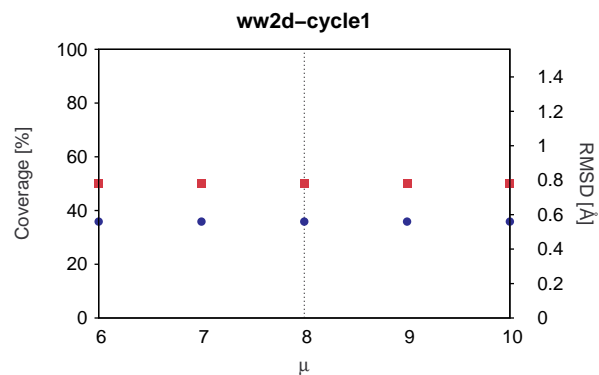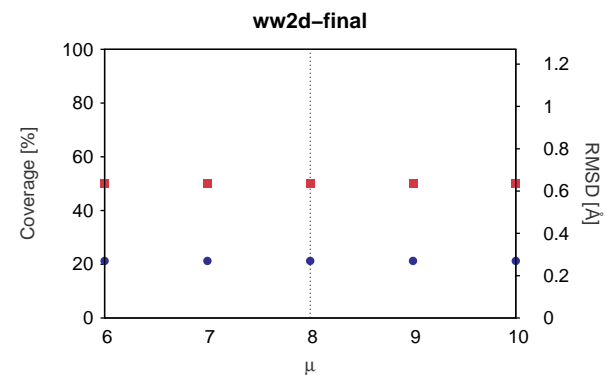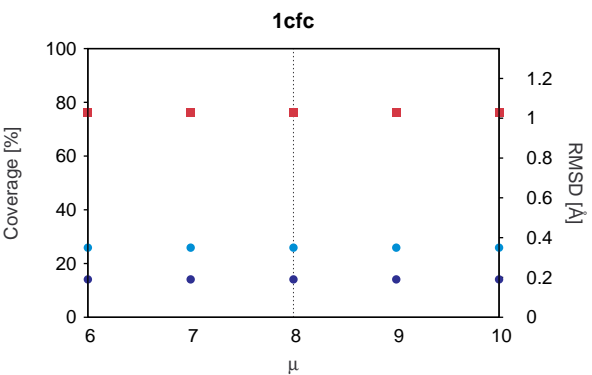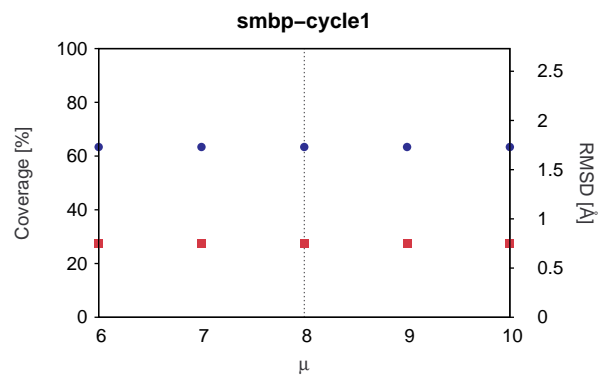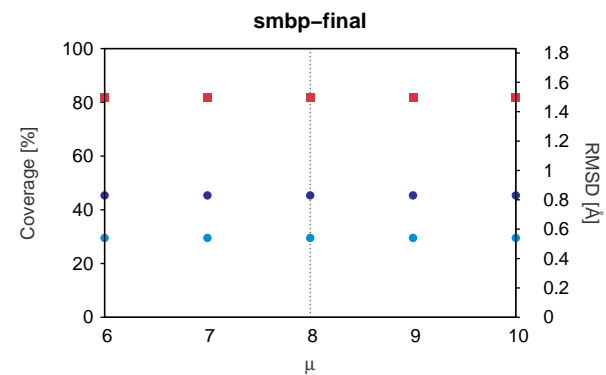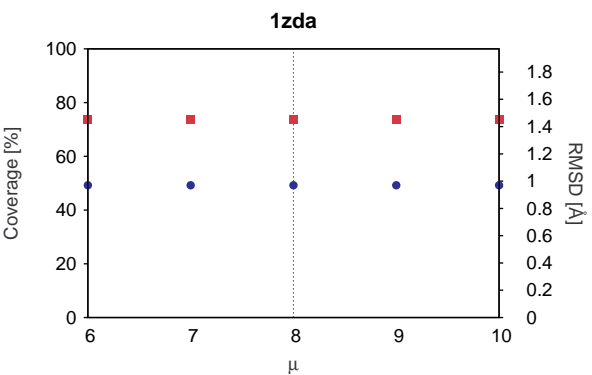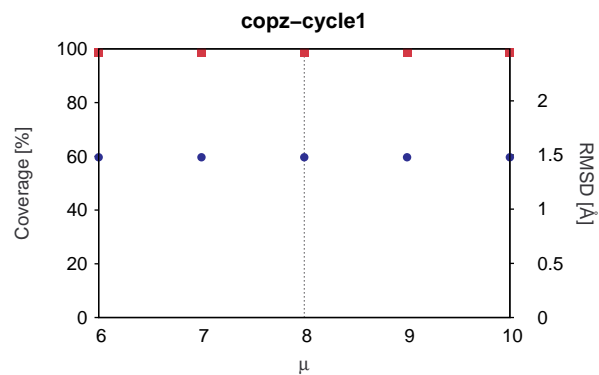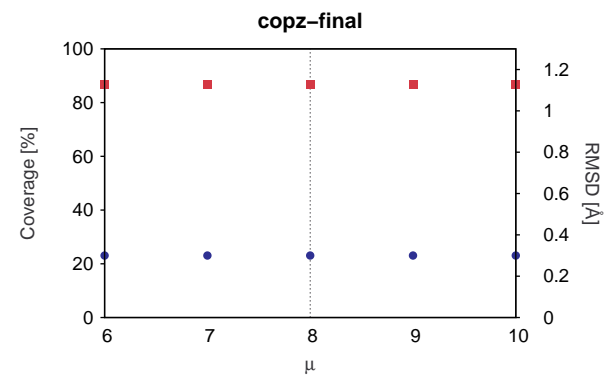

Supplement: Additional file 3 — Dependence of CYRANGE results on the minimal cluster size parameter μ. The sequence coverage (red) and RMSD (blue) of the residue ranges determined by CYRANGE were plotted as a function of μ for 9 different protein structure bundles. The dotted vertical line indicates the default value, μ = 8. Where CYRANGE found two domains, the RMSD values of the individual domains are shown in light and dark blue. [file 1471-2105-12-170-S3.PDF]

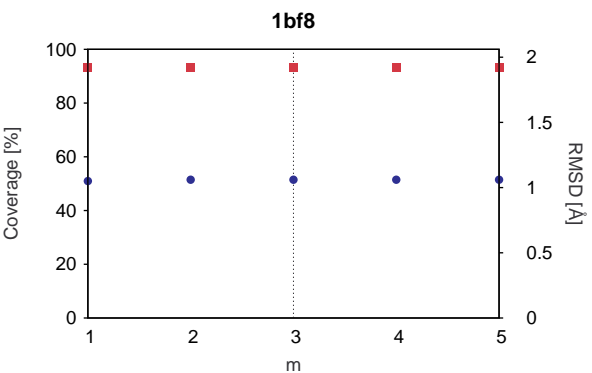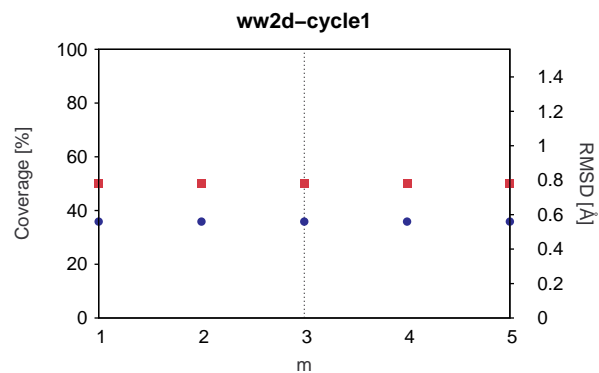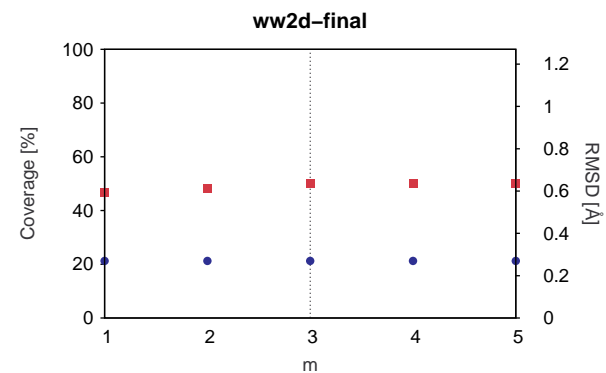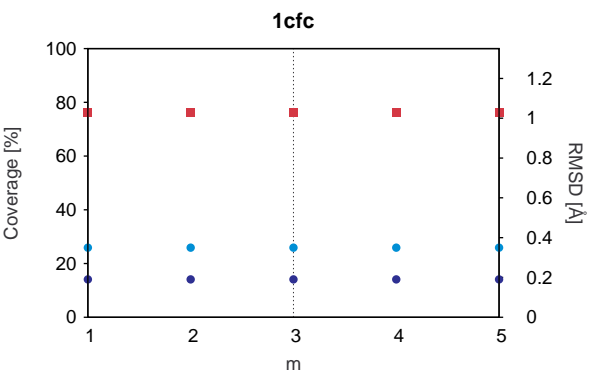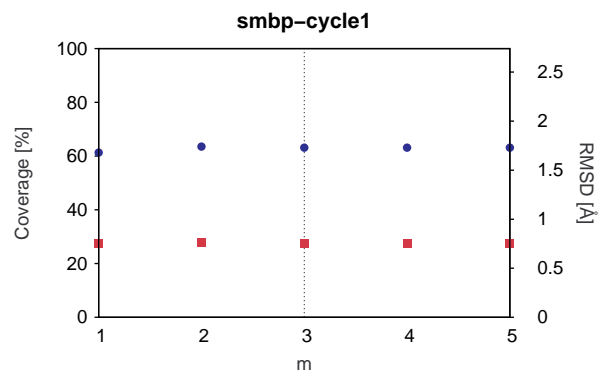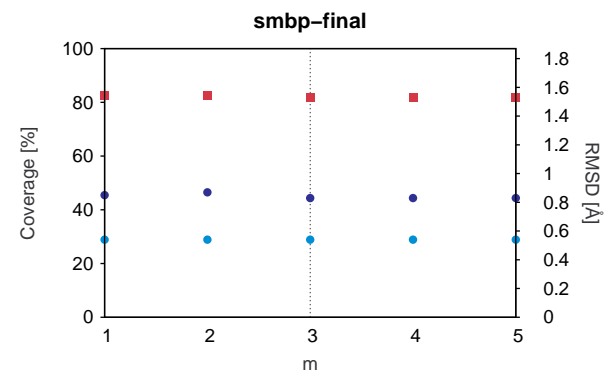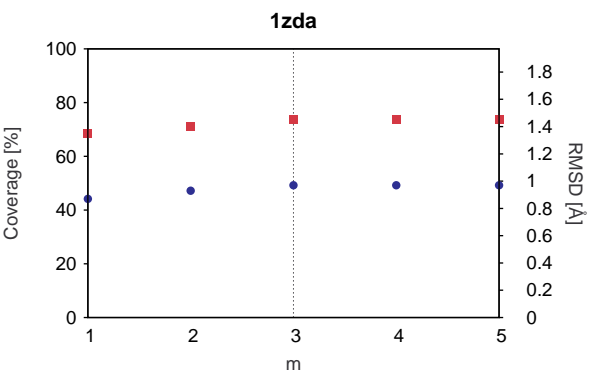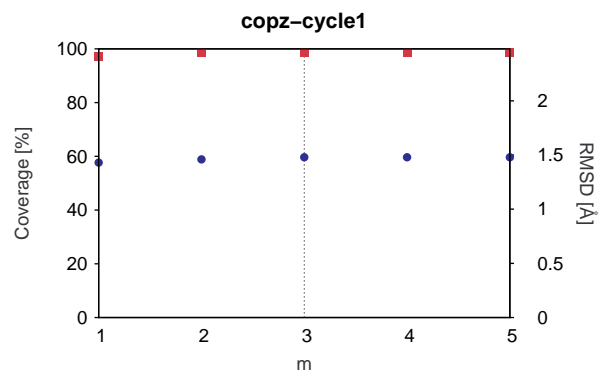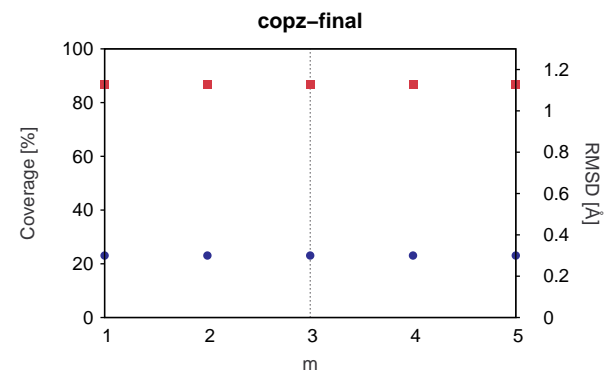

Supplement: Additional file 4 — Dependence of CYRANGE results on the domain boundary extension parameter m. See Additional File 3 for details. [file 1471-2105-12-170-S4.PDF]

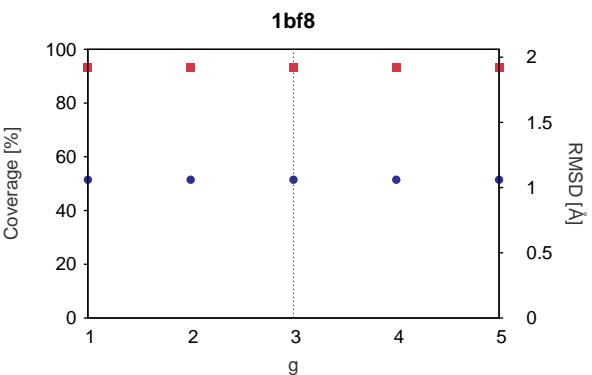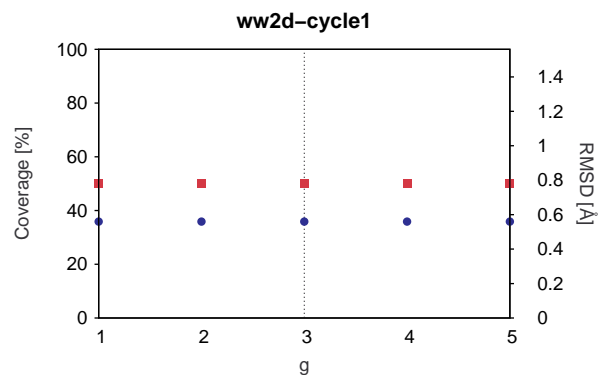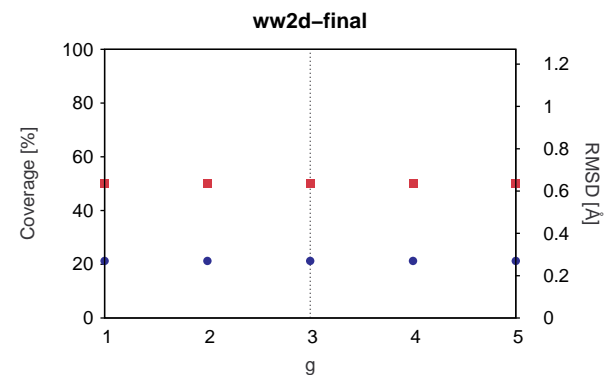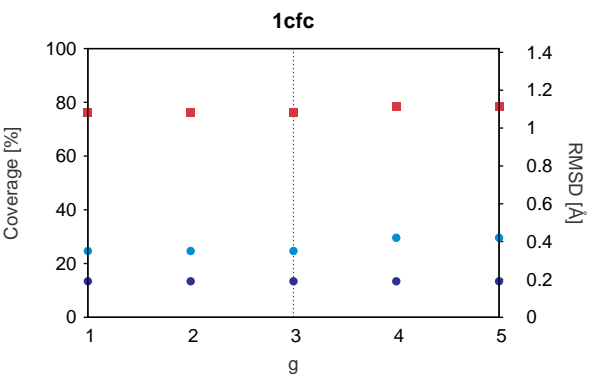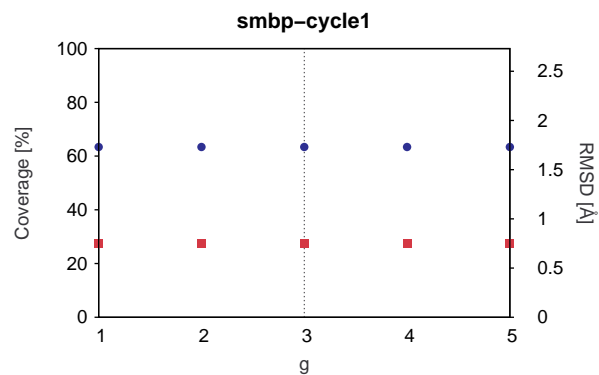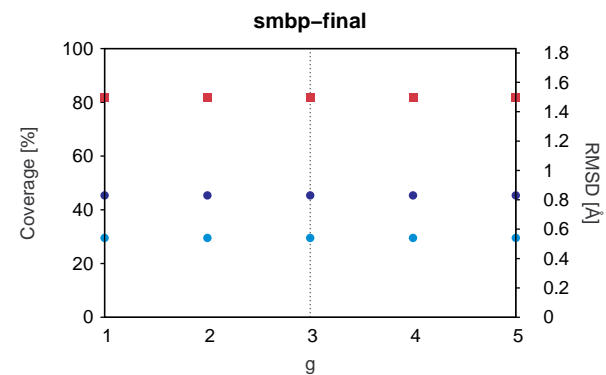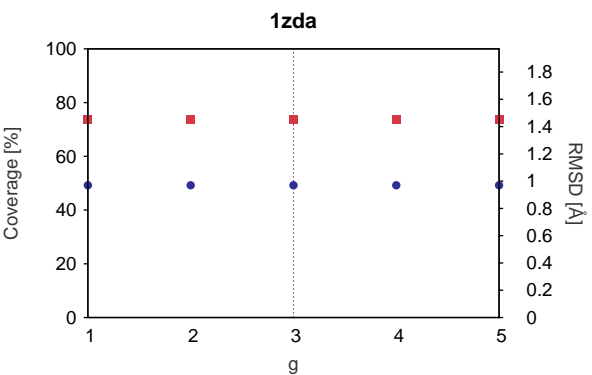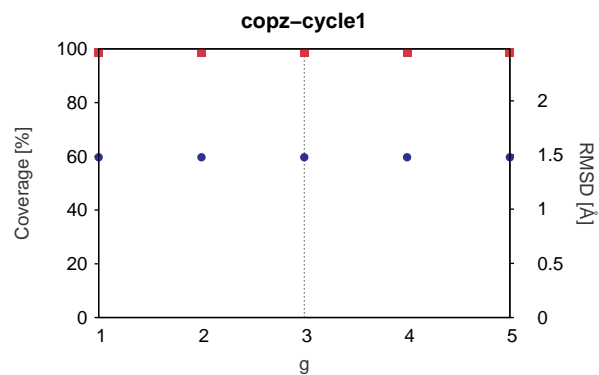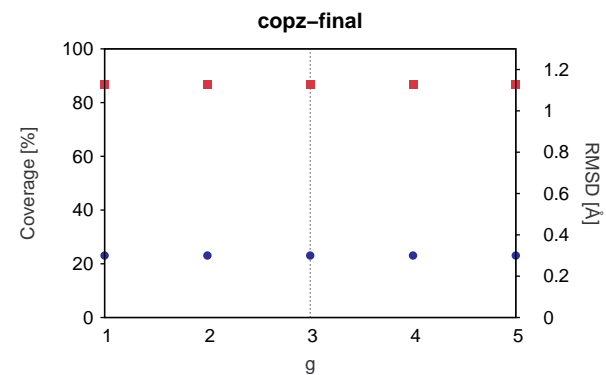

Supplement: Additional file 5 — Dependence of CYRANGE results on the minimal gap width g. See Additional File 3 for details. [file 1471-2105-12-170-S5.PDF]

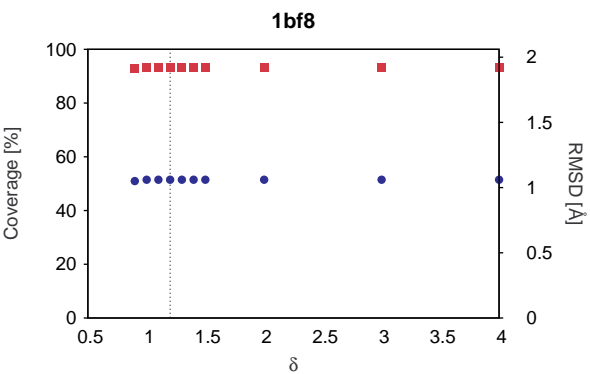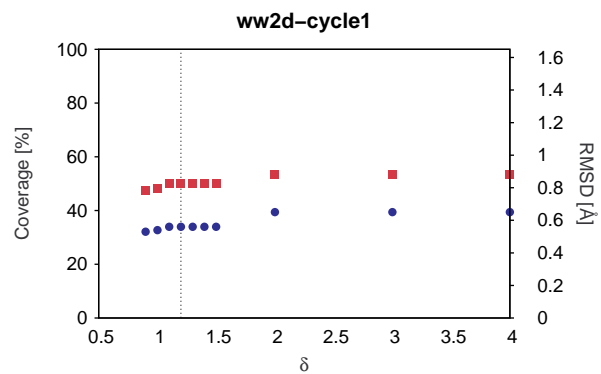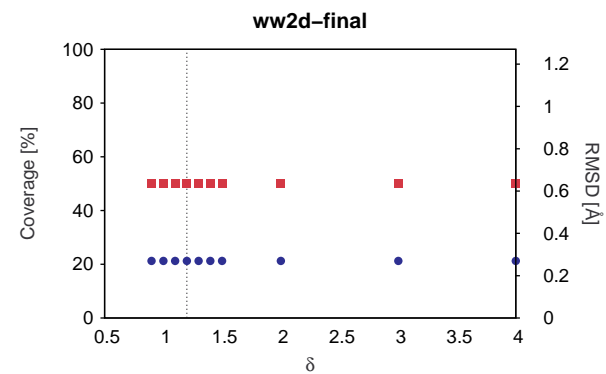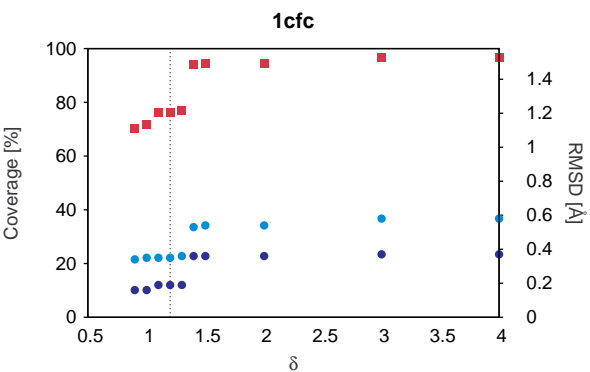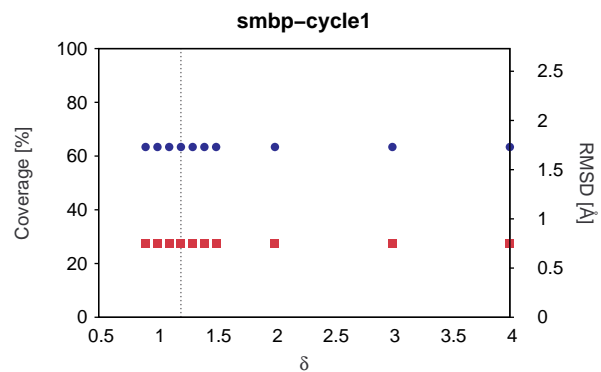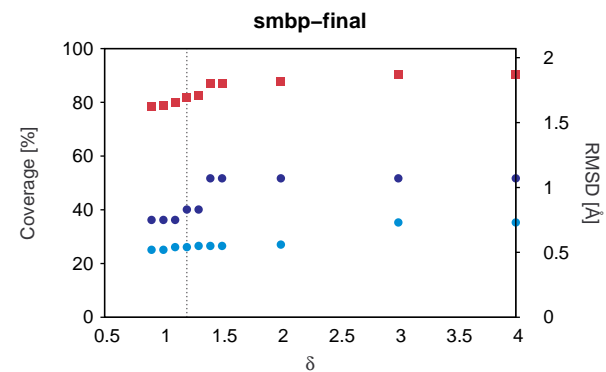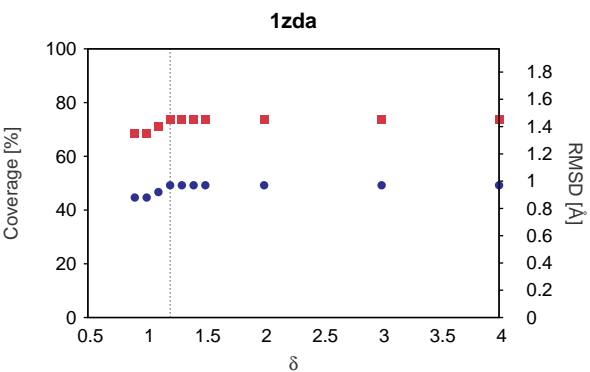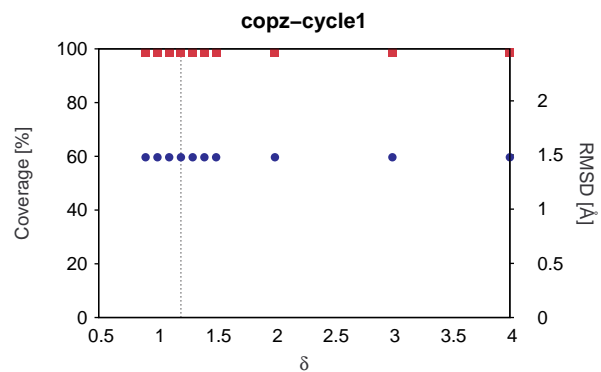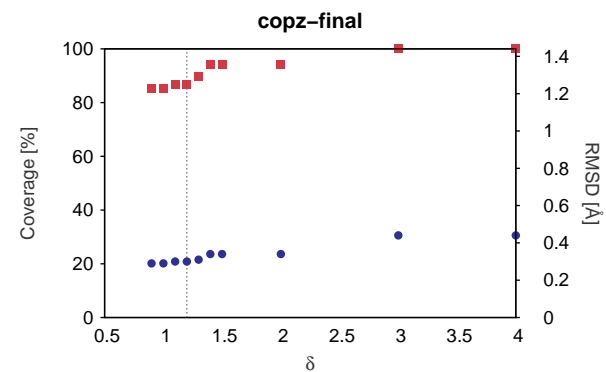

Supplement: Additional file 6 — Dependence of CYRANGE results on the relative RMSD decrease parameter δ. See Additional File 3 for details. [file 1471-2105-12-170-S6.PDF]

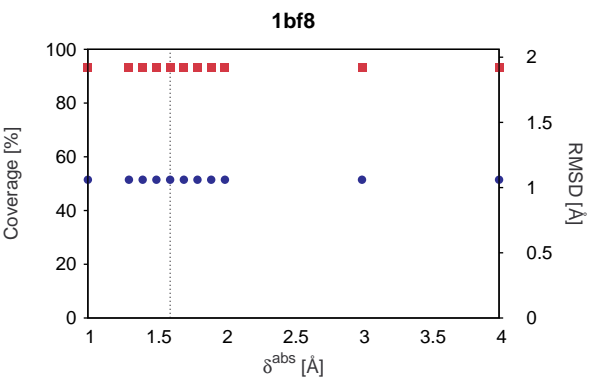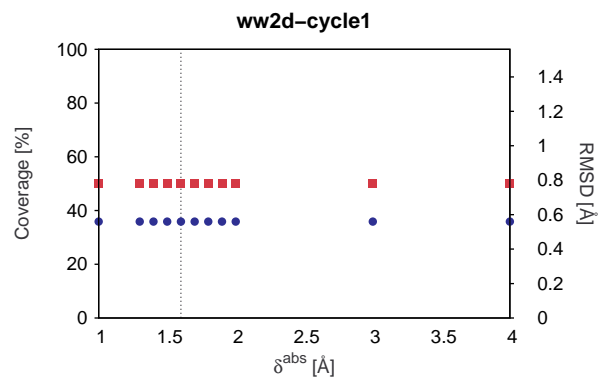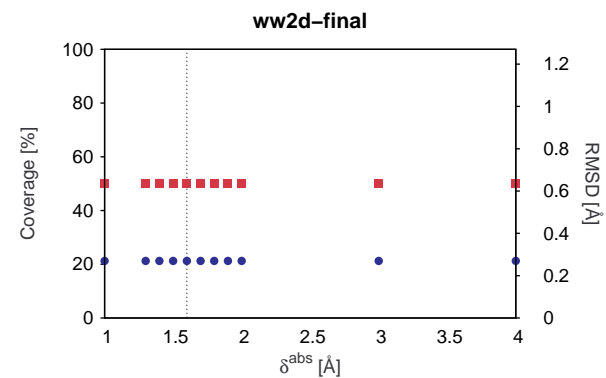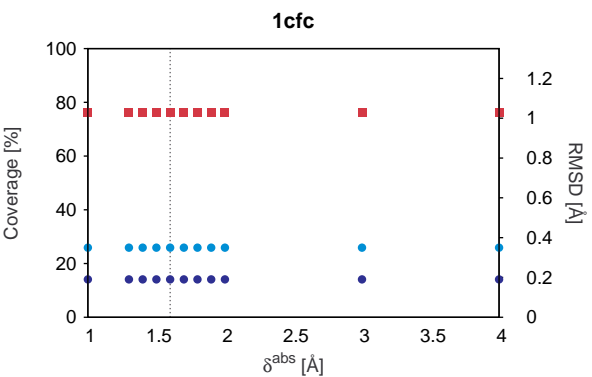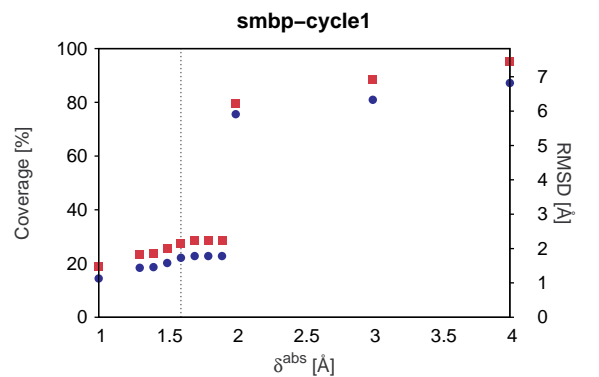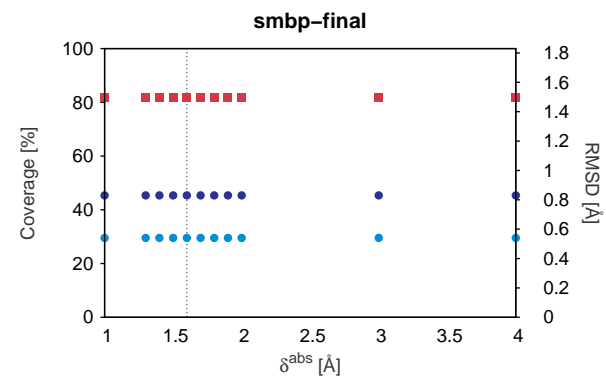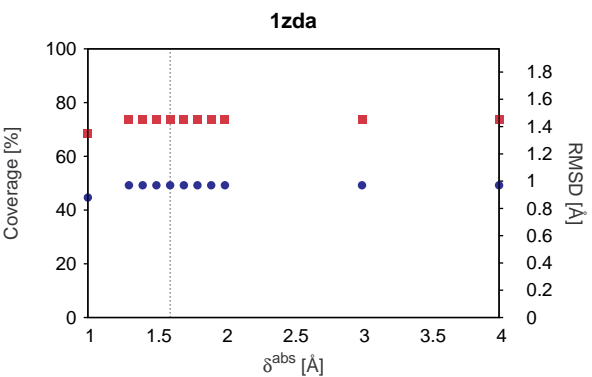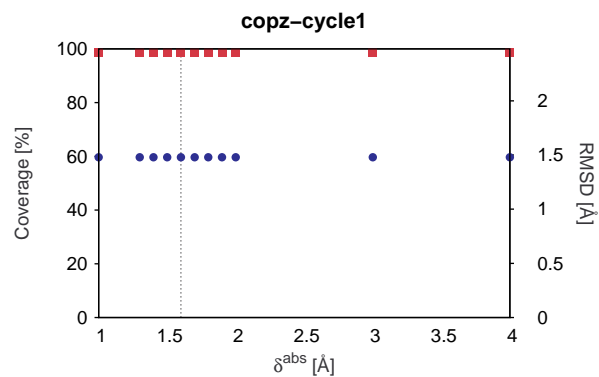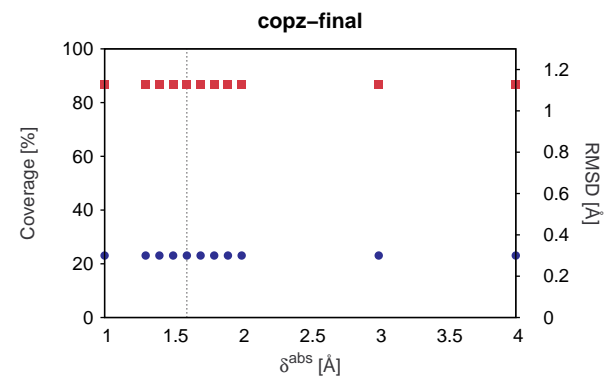

Supplement: Additional file 7 — Dependence of CYRANGE results on the absolute RMSD decrease parameter δ abs. See Additional File 3 for details. [file 1471-2105-12-170-S7.PDF]

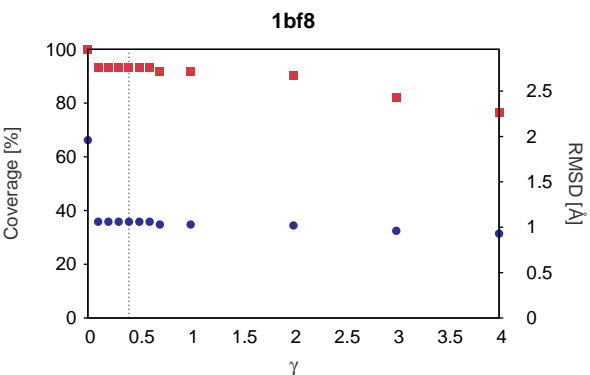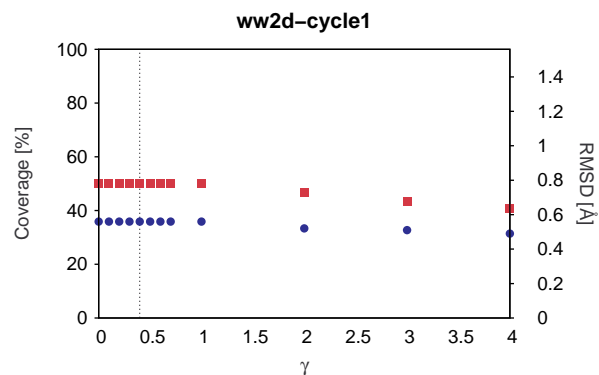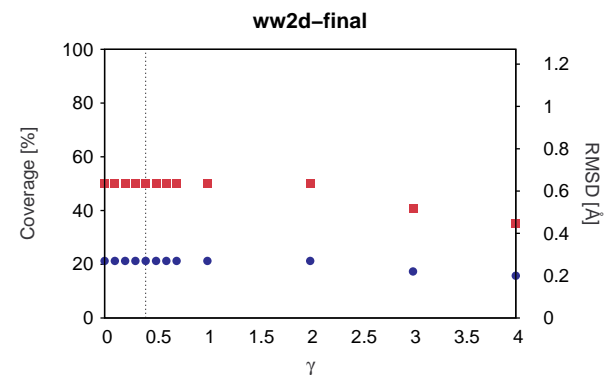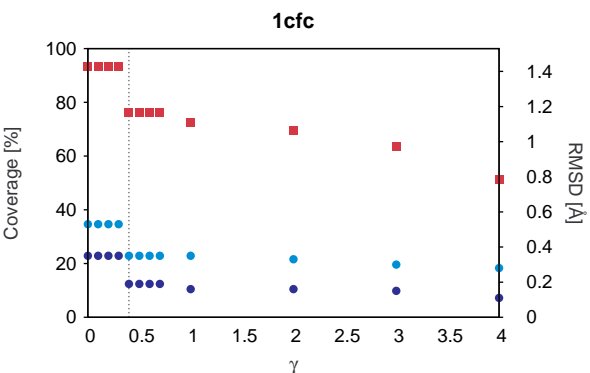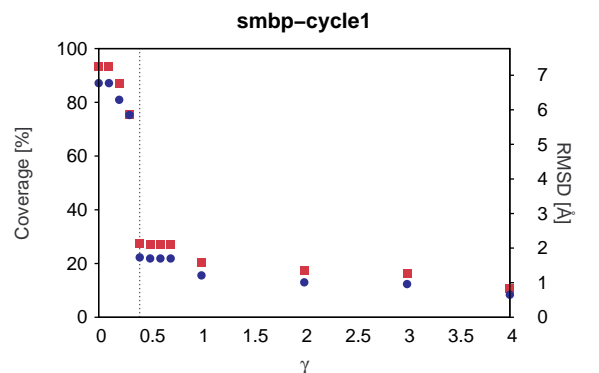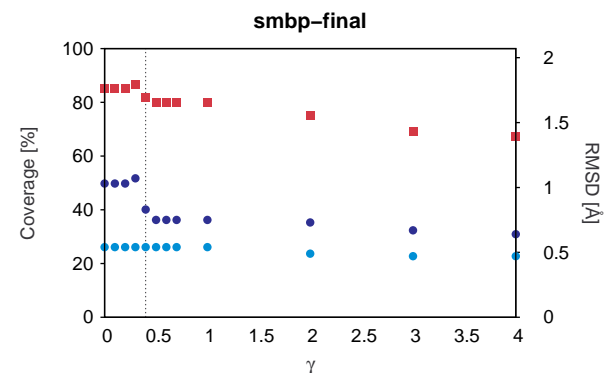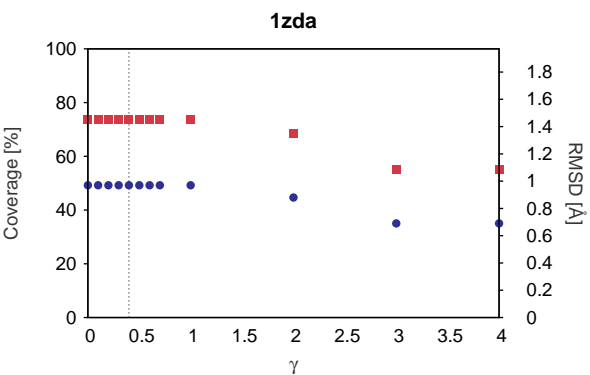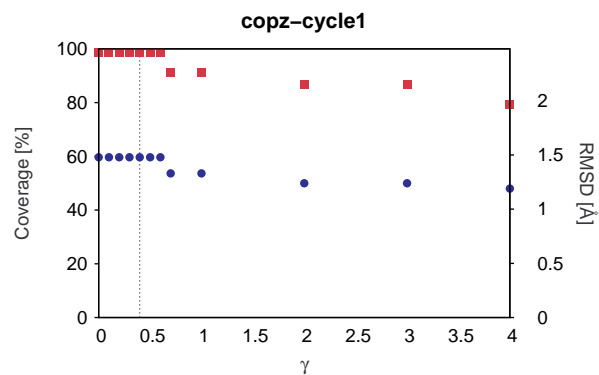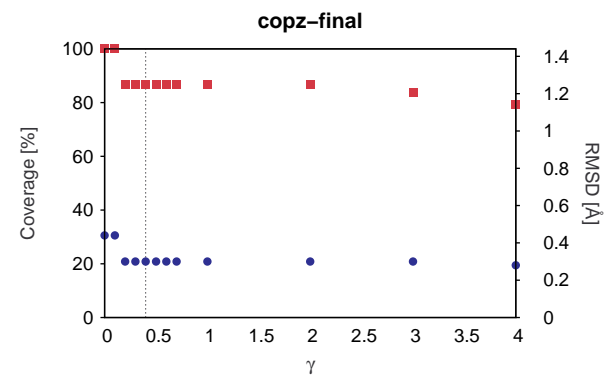

Supplement: Additional file 8 — Dependence of CYRANGE results on the gap penalty parameter γ. See Additional File 3 for details. [file 1471-2105-12-170-S8.PDF]

**CYRANGE**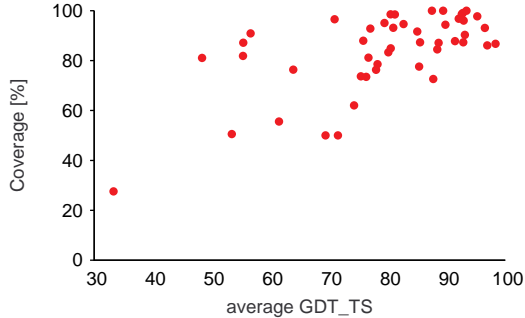**FindCore**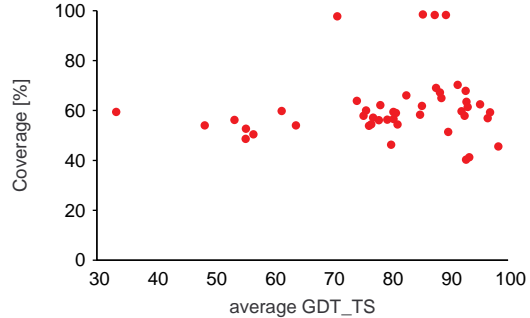**PSVS**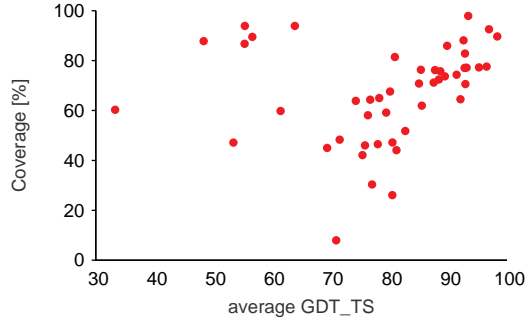

Supplement: Additional file 9 — Correlation between the sequence coverage from CYRANGE, FindCore and PSVS, and the GDT total score, GDT_TS. Each data point represents a protein shown in Figures 3 and 4. The coverage is the percentage of amino acid residues included in the residue ranges found by the different methods. The GDT_TS value is defined by GDT_TS = (P1 + P2 + P4 + P8)/4, where Pd is the fraction of residues that can be superimposed under a distance cutoff of d Å. [file 1471-2105-12-170-S9.PDF]

**CYRANGE**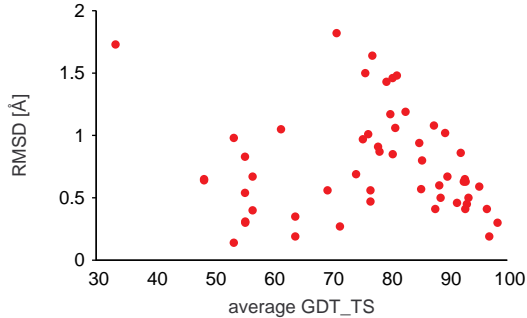**FindCore**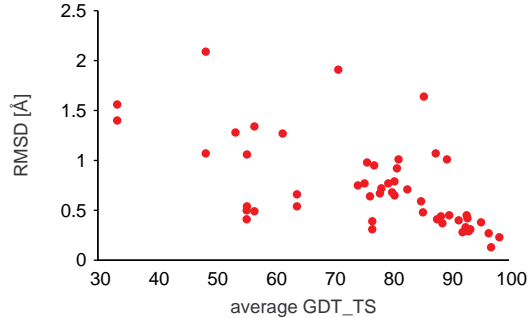**PSVS**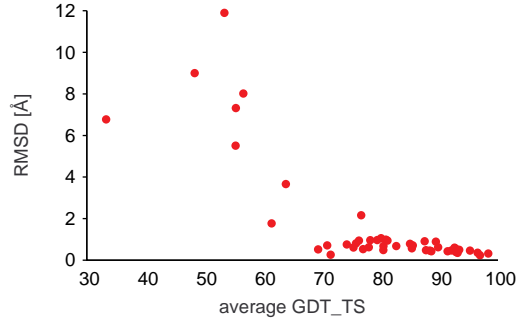

Supplement: Additional file 10 — Correlation between the RMSD value for the residue ranges from CYRANGE, FindCore and PSVS, and the GDT total score, GDT_TS. Each data point represents one protein domain. See Additional File 9 for details. [file 1471-2105-12-170-S10.PDF]
